# Discovery of a First-in-Class Covalent Allosteric SHP1 Inhibitor with Immunotherapeutic Activity

**DOI:** 10.1002/anie.202525126

**Published:** 2025-12-26

**Authors:** Zihan Qu, Frederick Nguele Meke, Zheng Zhang, Aaron D. Krabill, Christine S. Muli, Brenson A. Jassim, Jiajun Dong, Quyen D. Nguyen, Yunpeng Bai, Jinyue Li, Yiming Miao, Bardia Asadi, Levi M. Johnson, Jinmin Miao, Darci J. Trader, W. Andy Tao, Zhong-Yin Zhang

**Affiliations:** James Tarpo Jr. and Margaret Tarpo Department of Chemistry, Purdue University, 560 Oval Drive, West Lafayette, Indiana 47907, USA; Borch Department of Medicinal Chemistry and Molecular Pharmacology, Purdue University, 575 Stadium Mall Drive, West Lafayette, Indiana 47907, USA; Department of Biochemistry, Purdue University, 175 South University Street, West Lafayette, Indiana 47907, USA; Borch Department of Medicinal Chemistry and Molecular Pharmacology, Purdue University, 575 Stadium Mall Drive, West Lafayette, Indiana 47907, USA; Borch Department of Medicinal Chemistry and Molecular Pharmacology, Purdue University, 575 Stadium Mall Drive, West Lafayette, Indiana 47907, USA; Borch Department of Medicinal Chemistry and Molecular Pharmacology, Purdue University, 575 Stadium Mall Drive, West Lafayette, Indiana 47907, USA; Borch Department of Medicinal Chemistry and Molecular Pharmacology, Purdue University, 575 Stadium Mall Drive, West Lafayette, Indiana 47907, USA; James Tarpo Jr. and Margaret Tarpo Department of Chemistry, Purdue University, 560 Oval Drive, West Lafayette, Indiana 47907, USA; Borch Department of Medicinal Chemistry and Molecular Pharmacology, Purdue University, 575 Stadium Mall Drive, West Lafayette, Indiana 47907, USA; James Tarpo Jr. and Margaret Tarpo Department of Chemistry, Purdue University, 560 Oval Drive, West Lafayette, Indiana 47907, USA; Borch Department of Medicinal Chemistry and Molecular Pharmacology, Purdue University, 575 Stadium Mall Drive, West Lafayette, Indiana 47907, USA; Borch Department of Medicinal Chemistry and Molecular Pharmacology, Purdue University, 575 Stadium Mall Drive, West Lafayette, Indiana 47907, USA; James Tarpo Jr. and Margaret Tarpo Department of Chemistry, Purdue University, 560 Oval Drive, West Lafayette, Indiana 47907, USA; Borch Department of Medicinal Chemistry and Molecular Pharmacology, Purdue University, 575 Stadium Mall Drive, West Lafayette, Indiana 47907, USA; Borch Department of Medicinal Chemistry and Molecular Pharmacology, Purdue University, 575 Stadium Mall Drive, West Lafayette, Indiana 47907, USA; James Tarpo Jr. and Margaret Tarpo Department of Chemistry, Purdue University, 560 Oval Drive, West Lafayette, Indiana 47907, USA; Department of Biochemistry, Purdue University, 175 South University Street, West Lafayette, Indiana 47907, USA; Institute for Cancer Research, Purdue University, 201 South University Street, West Lafayette, Indiana 47907, USA; Institute for Drug Discovery, Purdue University, 720 Clinic Drive, West Lafayette, Indiana 47907, USA; James Tarpo Jr. and Margaret Tarpo Department of Chemistry, Purdue University, 560 Oval Drive, West Lafayette, Indiana 47907, USA; Borch Department of Medicinal Chemistry and Molecular Pharmacology, Purdue University, 575 Stadium Mall Drive, West Lafayette, Indiana 47907, USA; Institute for Cancer Research, Purdue University, 201 South University Street, West Lafayette, Indiana 47907, USA; Institute for Drug Discovery, Purdue University, 720 Clinic Drive, West Lafayette, Indiana 47907, USA

**Keywords:** Allosteric inhibition, Covalent inhibitors, Immunotherapy, Ligandable cysteine, Protein tyrosine phosphatases

## Abstract

Src homology 2 domain-containing phosphatase 1 (SHP1), encoded by *PTPN6*, is a key intracellular mediator of inhibitory immune signals. SHP1 is garnering attention as a potential immunotherapeutic target since SHP1 deletion elicits strong antitumor activity by boosting both innate and adaptive immunity. Unfortunately, no quality SHP1 inhibitor exists to demonstrate its translatability owing to the challenges posed by the chemistry of the phosphatase active site. Herein, we describe the discovery of a first-in-class, phenyl chloroacetamide-based covalent allosteric SHP1 inhibitor M029 through covalent fragment screening and multiparameter optimization. M029 inactivates SHP1 by covalently binding to a non-conserved and cryptic Cys480 far away from the active site, thus uncovering a novel allosteric mechanism for SHP1 inhibition. In addition, M029 is highly selective for SHP1 and exhibits robust cellular target engagement. Importantly, M029 is orally active and blocks tumor progression in a syngeneic cancer model by activating natural killer cells and cytotoxic CD8^+^ T cells, along with reduced T cell exhaustion. Together, this study reveals a ligandable Cys that can be exploited for allosteric inhibition of SHP1, which has been refractory to targeted pharmacologic manipulation. The work also demonstrates small-molecule SHP1 inhibition as a compelling approach for new cancer immunotherapy.

## Introduction

Immunotherapy, which harnesses the body’s immune system to identify and eradicate malignant cells, has become a prominent therapeutic modality for cancer treatment.^[1,2]^ Current immunotherapeutic approaches generally fall into two categories: immune checkpoint blockade and cell-based therapies.^[3]^ Antibodies that target immune checkpoint receptors, such as programmed death 1 (PD-1) or cytotoxic T lymphocyte antigen 4 (CTLA-4), have brought significant therapeutic benefits to cancer patients.^[4–6]^ Adoptive cell therapies using chimeric antigen receptor T cells (CAR-T) have revolutionized the treatment of hematological malignancies.^[7]^ Nonetheless, the majority of cancer patients are still refractory to existing immunotherapies. In addition to substantial expenses incurred, contemporary immunotherapies are also often associated with significant immunerelated adverse events.^[8]^ Furthermore, since most of the aforementioned immunotherapies target surface antigens, they are susceptible to antigen loss and exhibit limited efficacy in tumors with low antigen presentations, along with inadequate penetration to the tumor microenvironment.^[9,10]^ Consequently, there is a clear need to find new targets and develop novel approaches to improve cancer immunity and benefit more cancer patients. To that end, the pursuit of small-molecule immune modulators addressing intracellular targets and pathways has emerged as an increasingly attractive avenue for immunotherapy.^[11–13]^ Small molecule agents can access intracellular targets, be orally bioavailable, and reach all organs with greater tissue/tumor penetration.^[11,14,15]^ Small molecule dosages are more amenable to fine control of their plasma concentration and circumvent immunerelated adverse events experienced with long-lasting biologics. Finally, small molecules can be produced at much lower cost, thus enabling greater patient access.^[11]^

Protein tyrosine phosphatases (PTPs) are essential for tyrosine phosphorylation-mediated signal transduction that controls numerous cellular processes, including growth, survival, and the immune response.^[16,17]^ A number of PTPs have been identified as negative regulators of immune cells, among which SHP1 (encoded by *PTPN6*) holds considerable significance as a promising target for the development of small-molecule immunotherapeutic agents.^[18–20]^ SHP1 is predominantly expressed in all hematopoietic lineages and functions as a negative regulator of the immune system.^[21,22]^ Mice harboring spontaneous mutations in *PTPN6* develop inflammatory skin diseases and autoimmunity,^[23–27]^ while heterozygous missense and splice variant mutations in *PTPN6* have also been found in human patients with neutrophilic dermatoses and emphysema, accompanied by the manifestation of autoimmune symptoms.^[28,29]^ These observations thereby underscore the negative regulatory role of SHP1 in immunity. Importantly, SHP1 deficiency in T cells lowers the activation threshold, increases the production of effector CD8^+^ T cells, renders CD8^+^ and CD4^+^ T cells resistant to regulatory T cell (T_reg_)-mediated suppression, and culminates in the accumulation of memory T cells.^[30–33]^ Additionally, SHP1 abrogation in adoptively transferred T cells enhances antitumor efficacy and potentiates PD-1 and CTLA-4 immune checkpoint blockades.^[34,35]^ Furthermore, SHP1 activity is also needed for setting the threshold of natural killer (NK) cell reactivity,^[36]^ and SHP1-deficient NK cells exhibit enhanced tumoricidal capacity.^[37–39]^ Besides NK cells, loss of SHP1 also promotes macrophage antitumor immunity.^[40]^ Finally, global inducible deletion of SHP1 led to robust antitumor immunity in two syngeneic tumor models, MC38 (colon cancer) and EO771 (breast cancer).^[40]^ Collectively, these studies indicate that SHP1 transduces inhibitory signals downstream of immunoreceptors in immune cells and suggest that targeting SHP1 with small-molecule inhibitors may be an attractive immunotherapeutic strategy to unleash both innate and adaptive immunity against tumor cells.

Given the large body of biochemical and genetic evidence bolstering SHP1 as a promising target for immunotherapy, there is heightened interest in SHP1 inhibition as a new therapeutic approach for cancer treatment. Moreover, SHP1 may also play tumor-suppressive roles in certain hematologic and solid malignancies, whereas in some other cancer types it can exert a tumor-promoting effect.^[41]^ Therefore, it is evident that we do not understand the full spectrum of cell signaling regulation by SHP1 and how it stimulates or suppresses tumorigenesis. Thus, potent and selective SHP1 inhibitors could serve as valuable tools to interrogate the functions of SHP1 in health and diseases. Unfortunately, no high-quality SHP1 inhibitor exists to establish the translatability of this target. Here, we describe the discovery and characterization of a first-in-class phenyl chloroacetamide-based covalent allosteric SHP1 inhibitor M029, which targets a cryptic non-active site Cys residue in SHP1 and allosterically blocks SHP1 phosphatase activity. In addition, M029 is highly selective for SHP1 and exhibits robust cellular target engagement. Moreover, M029 specifically attenuates SHP1-dependent signaling and enhances T-cell activation. Importantly, M029 is orally efficacious and suppresses tumor progression in mice bearing syngeneic MC38 tumors through increased T cell and NK cell infiltration and activation. Our findings present a novel strategy for SHP1 inhibition by targeting a cryptic allosteric Cys, thereby circumventing the challenges posed by the chemistry of the PTP active site. The study also demonstrates small-molecule SHP1 inhibition as a compelling path for new cancer immunotherapy and offers a potential lead that can be further developed for clinical translation.

## Results and Discussion

### Discovery of Covalent SHP1 Inhibitors

As noted above, the ability to selectively stimulate cancer immunity through SHP1 inhibition holds enormous therapeutic potential. However, despite its known roles in immune cells, SHP1 remains unexplored as a therapeutic target due to the absence of small molecule inhibitors with the requisite potency, selectivity, and drug-like properties for target interrogation and clinical translation. Given the challenging nature of drugging the PTP active site,^[42–44]^ we focused on the discovery of ligands that bind covalently to SHP1. Targeted covalent inhibitors, which make a covalent bond with the target protein for irreversible inactivation, offer several benefits when compared to traditional reversible non-covalent inhibitors, including prolonged target engagement, the ability to outcompete high-affinity substrates, potential for increased selectivity within homologous proteins by modifying non-conserved residues, and the capability to target shallow binding sites.^[45–50]^ The many benefits of covalency have led to a renaissance in medicinal chemistry to explore the covalent drug space despite concerns about their chemical reactivity. Recent clinical successes of covalent kinase and KRAS/G12C inhibitors have prompted a renewed interest in developing covalent inhibitors for drug discovery.^[51,52]^

The PTP-catalyzed reaction involves a nucleophilic attack by the conserved active site Cys side chain on the phosphorus atom of phosphotyrosine in the substrate.^[53]^ As such, the PTPs could serve as prime targets for covalent inhibition. Previously described activity-based probes or covalent ligands for the PTPs are either phosphotyrosine isosteres or thiolreactive compounds that covalently modify the active site residues (including the catalytic Cys) and lack sufficient isozyme specificity.^[54–61]^ Inspired by the resurgence in covalent inhibitors for difficult drug targets, we set out to discover covalent SHP1 inhibitors by screening for electrophilic fragments that bind covalently to SHP1. This strategy is increasingly utilized to identify novel ligands for a target of interest.^[47,62,63]^ We assembled a library of 4600 Cys reactive molecules from the Enamine covalent fragment collections containing 49.0% acrylamide, 38.1% chloroacetamide, 5.15% chloromethylketone, 5.20% activated nitrile, 2.10% epoxide, and 0.53% disulfide (Figure S1A). We carried out a high-throughput biochemical fragment screening of this curated commercially available library against the SHP1 catalytic domain (residues 245–543). Each fragment (500 μM) was incubated with SHP1 (500 nM) for 30 min at room temperature before diluting the reaction 5.5-fold with 24.45 mM para-nitrophenyl phosphate (*p*NPP) to measure the residual PTP activity. This concentration of substrate was chosen to out-compete any remaining fragment to reduce the inactivation of SHP1 during the enzymatic hydrolysis of *p*NPP. After a 2-min incubation at 25 °C, the reaction was quenched by the addition of 5N NaOH, and the production of para-nitrophenol was determined by absorbance at 405 nm. This assay format afforded a Z’-factor of 0.70 (Figure S1B), indicating a robust screen to reliably identify hit molecules. To enhance the probability of discovering covalent ligands with inherent preference for SHP1, we also counter-screened the same library against SHP2, the closest homolog of SHP1, and two other widely studied PTPs, PTP1B and PTPN22 (also called LYP). From this screening campaign, we identified 297 hit molecules that displayed greater than 50% SHP1 inhibition. Among these, 31 were repurchased based on their observed preference for SHP1 and structural diversity, 11 of which were confirmed as potential covalent inhibitors for SHP1 based on the time dependency of their half-maximal inhibition concentration (IC_50_) values determined with a 10-min and 60-min preincubation time, respectively (Table 1).

**Figure 1.**
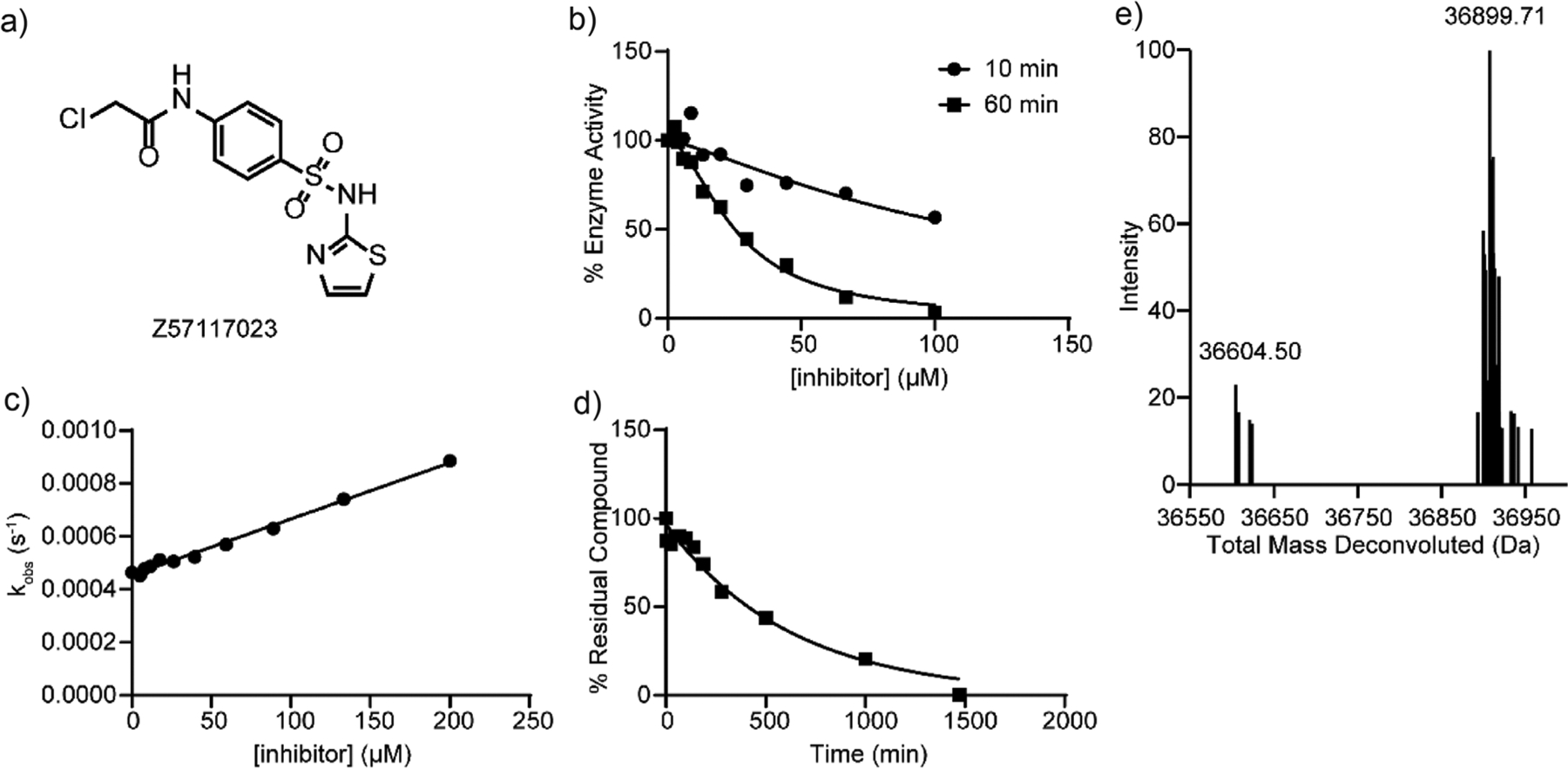
Primary hit Z57117023 is a selective SHP1 covalent inhibitor. a) Chemical structure of Z57117023. b) Z57117023 showed an IC_50_ of 117.6 ± 8.3 μM after 10 min preincubation with SHP1 and 25.2 ± 1.6 μM after 60 min preincubation with SHP1. The increased potency with prolonged incubation further supported the time-dependence of SHP1 inhibition by Z57117023. c) The inactivation *k*_inact_/*K*_I_ of Z57117023 was determined to be 227.5 M^−1^ s^−1^ using a time-course pNPP assay, demonstrating its dose- and time-dependent inhibition of SHP1. d) Z57117023 was incubated with a 100-fold excess of GSH in 3,3-dimethylglutarate buffer (pH = 7.0) at room temperature, and its half-life was determined to be 424.0 ± 55.1 min using LC-MS. The moderate half-life in the presence of excessive GSH implied the low intrinsic reactivity of Z57117023. e) SHP1 protein was incubated with or without Z57117023 in 3,3-dimethylglutarate buffer (pH = 7.0) for 60 min at room temperature to form a covalent adduct. The mixture was characterized by LC-MS, where Z57117023-treated SHP1 protein showed a single adduct with a mass shift of 295.21 Da, corresponding to the molecular weight of Z57117023 adduct with the chlorine displaced, confirming its target engagement and mode of action.

**Scheme 1.**
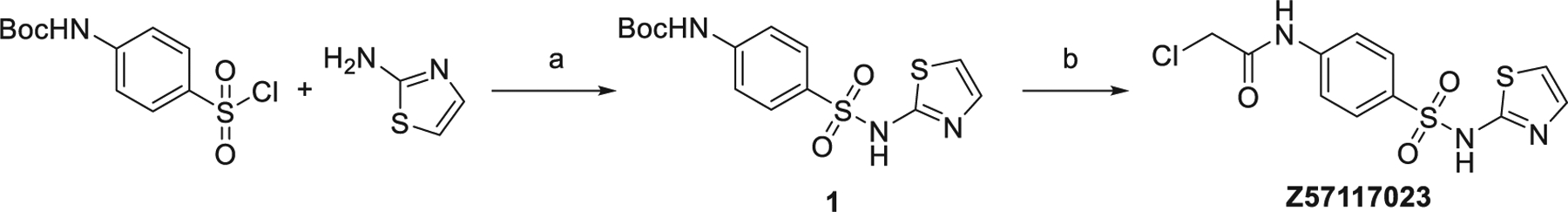
Synthesis of hit Z57117023. Reagents and conditions: a) pyridine, toluene, 65 °C, overnight. b) (i) TFA, DCM, rt, overnight. (ii) chloroacetyl chloride, DCM, 0 °C then rt, 6 h.

To ensure that these putative SHP1 covalent ligands are not promiscuously reactive molecules that lack target selectivity, we also assessed their inherent reactivity toward thiols using glutathione (GSH) as a reference.^[62,64–66]^ One of the 11 validated hits, Z57117023 (Figure 1a), a phenyl chloroacetamide with a terminal thiazole ring tethered by a sulfonamide linker, exhibited > 11-fold selectivity for SHP1 over SHP2, PTP1B, and PTPN22, and displayed minimal reactivity toward GSH. Given its selectivity for SHP1 and chemical stability, compound Z57117023 was prioritized for additional characterization. To further validate its SHP1 inhibitory activity, we resynthesized Z57117023 as depicted in Scheme 1. In short, Z57117023 was prepared via the sulfonylation of 2-aminothiazole by commercially available *tert*-butyl (4-(chlorosulfonyl)phenyl)carbamate, followed by the removal of the Boc protecting group and subsequent amide formation with chloroacetyl chloride. The resynthesized Z57117023 exhibited time-dependent SHP1 inhibition and comparable IC_50_ values to those of the purchased compound (Figure 1b and Table 1), confirming Z57117023 as a potential covalent inhibitor of SHP1.

Covalent inhibitors generally inactivate their targets through a two-step process, starting with a reversible binding event (characterized by the dissociation constant *K*_I_) and followed by covalent bond formation (represented by the first-order rate constant of inactivation *k*_inact_). Although IC_50_ values are often utilized to assess the potency of covalent inhibitors, the second-order rate constant *k*_inact_/*K*_I_ is preferred because it provides a measure of covalent inhibition in a time- and dose-independent manner.^[60,62,67,68]^ Because *k*_inact_/*K*_I_ measurement can be time and labor-intensive, we developed an efficient method to determine the observed first-order rate constant (*k*_obs_) from the analysis of a progress curve in the presence of an inhibitor.^[69]^ The *k*_obs_ value is then plotted as a function of inhibitor concentration to obtain the kinetic parameters *k*_inact_, *K*_I_, and *k*_inact_/*K*_I_. ^[70]^ Using this technique, we found that Z57117023 exhibits a *k*_inact_/*K*_I_ of 227.5 M^−1^ min^−1^ for SHP1 inactivation (Figure 1c). The reactivity of the resynthesized Z57117023 toward a general thiol group was further evaluated, and the compound displayed a half-life (t_1/2_) of approximately 7.1 ± 0.8 h toward excess GSH (Figure 1d). To verify SHP1 covalent modification by Z57117023, we ascertained SHP1 covalent adduct formation by intact protein liquid chromatography-mass spectrometry (LC-MS).^[58]^ A single SHP1-Z57117023 adduct was observed when SHP1 was incubated with Z57117023, with a mass shift of 295.21 Da (calculated 295.54 Da) (Figure 1e), consistent with the loss of a chlorine atom from Z57117023. Collectively, these results confirmed that Z57117023 is a genuine covalent inhibitor of SHP1 and that the mechanism by which it inactivates SHP1 involves the displacement of the chlorine from Z57117023 by a nucleophilic residue in SHP1.

### Acquisition and Characterization of M029 as a Covalent Allosteric Inhibitor of SHP1

As described above, Z57117023, a phenyl chloroacetamide, was identified as a covalent ligand for SHP1. Although chloroacetamides are less encountered in targeted covalent inhibition despite their comparable intrinsic reactivity to some of the commonly utilized covalent warheads (*e.g*., acrylamide),^[65]^ several chloroacetamide compounds have been shown to be efficacious in vivo.^[71–73]^ Given its observed preference for SHP1 and attenuated reactivity toward GSH, we surmised that Z57117023 serves as a promising chemical starting point for potent and selective SHP1 covalent inhibitor development. Our initial exploration of the structure-activity relationship (SAR) of SHP1 inhibition by Z57117023 was centered on a survey of its commercially available derivatives (Figure S2). This SAR “by-catalog” approach revealed that the parent and substituted phenyl chloroacetamides exhibited negligible SHP1 inhibitory activity even at 1 mM concentration with 60 min preincubation (Figure S2A). This observation suggests that the SHP1 inhibition by Z57117023 is dictated by its specific interactions with SHP1 in addition to the intrinsic reactivity of the phenyl chloroacetamide warhead. We next assessed Z57117023 derivatives containing the sulfonamide linker with various substituents. While phenyl chloroacetamindes bearing the sulfonamide linker with alkyl or a saturated 5- or 6-membered ring are inactive against SHP1 (Figure S2B), those with a benzene or pyridine displayed IC_50_ values for SHP1 inhibitory that were only 2.7 or 3.4-fold higher than that of Z57117023, which carries a thiazole substitution (Figure S2C). This indicates that the presence of a terminal aromatic moiety may be essential for Z57117023’s SHP1 inhibitory activity. Taken together, these findings highlight the importance of the phenyl chloroacetamide warhead, the sulfonamide linker, and the terminal aromatic ring structure for SHP1 inhibition, supporting the notion that Z57117023 holds great promise as a hit compound warranting subsequent optimization efforts.

**Figure 2.**
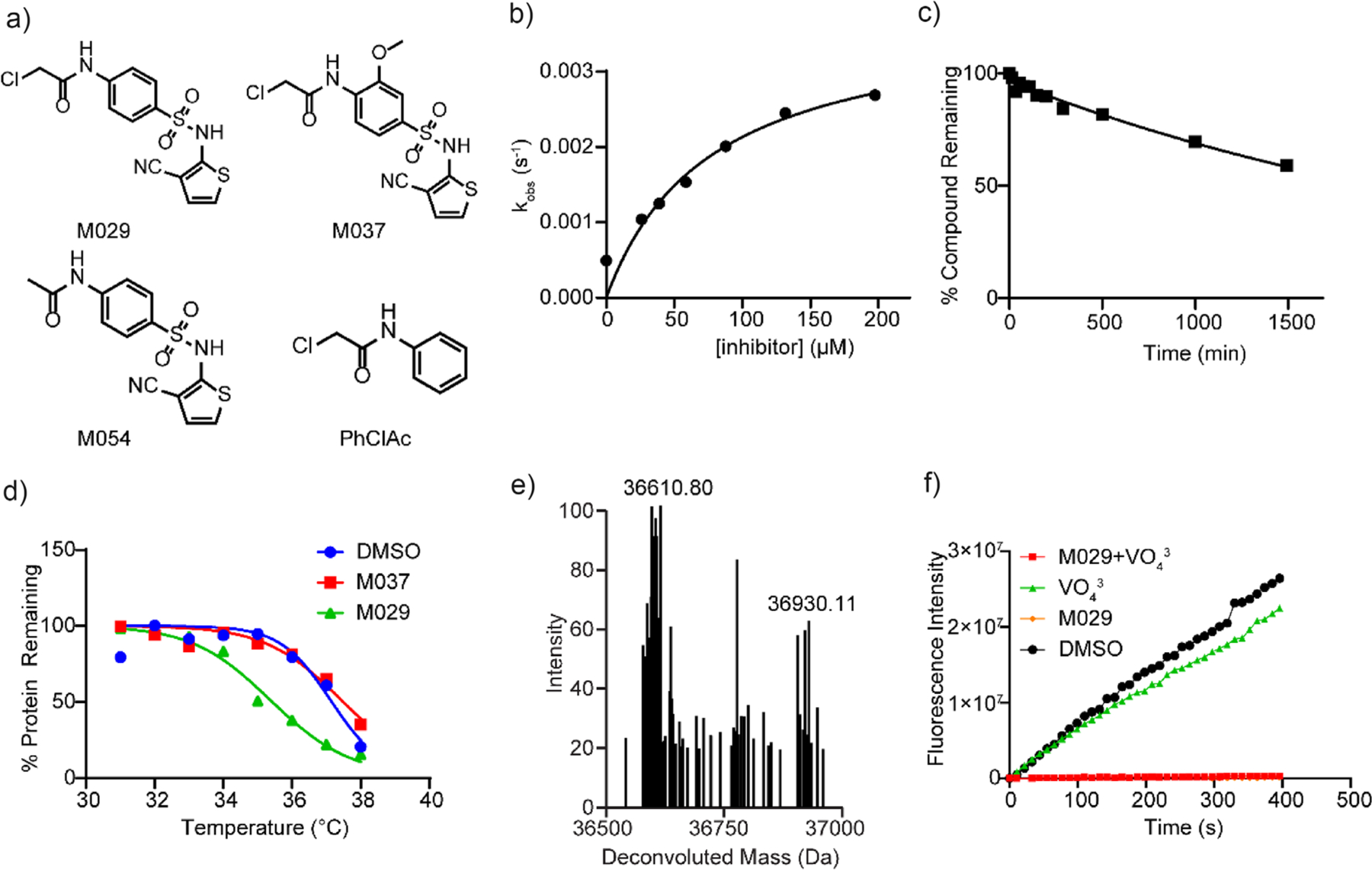
Characterization of SHP1 covalent inhibitor M029. a) The structures of M029 and its negative controls PhClAc, M037, and M054. b) The *k*_inact_ of M029 was determined to be 0.0038 ± 0.008 s^−1^, and the *K*_I_ was found to be 76.7 ± 12.5 μM at pH 7.0 and room temperature, yielding a *k*_inact_/*K*_i_ of 2973 M^−1^ min^−1^, which is 13-fold higher than that of Z57117023. c) M029 was incubated with a 100-fold excess of GSH in 3,3-dimethylglutarate buffer (pH = 7.0) at room temperature, and its half-life was determined to be 34.3 h using LC-MS, 5-fold longer than the parent compound Z57117023. d) SHP1 protein was incubated with M029, M037, or the DMSO control groups, followed by heating at different temperatures. The mixtures were centrifuged to remove the unfolded SHP1 proteins, and the supernatants were spiked with a control protein CDC14A, followed by loading into SDS-PAGE gels to quantify the relative SHP1 level. M029-treated SHP1 showed a 1.8 °C decrease in SHP1 melting point compared to the negative control and DMSO control, corroborating its covalent destabilization mechanism. e) SHP1 protein was incubated with or without M029 in 3,3-dimethylglutarate buffer (pH = 7.0) for 60 min at room temperature to form a covalent adduct. The mixture was characterized by LC-MS, where the M029-treated protein showed a single adduct with a mass shift of 319.31 Da, corresponding to the molecular weight of the M029 adduct with chlorine substituted. f) SHP1 protein was incubated with M029 with or without the pan-PTP competitive inhibitor sodium orthovanadate. The presence or absence of sodium orthovanadate did not show a significant difference in M029 inhibition against SHP1, suggesting that M029 occupies a different site from the SHP1 active site.

Building on the results from the SAR “by-catalog” study, the following medicinal chemistry effort was prioritized on a systematic survey of the central phenyl scaffold, the sulfonamide linker, and the distal aryl moiety with the goal of a multiparameter optimization of potency and selectivity for SHP1 inhibition as well as drug-like properties such as aqueous solubility and cell permeability. Additionally, given the crucial requirement for targeted covalent inhibitors to maintain tempered reactivity that enables selective target engagement and reduces cross-reactivity to by-standing nucleophiles in the cellular milieu,^[74,75]^ the medicinal chemistry campaign also sought to lower SHP1 covalent inhibitor reactivity with GSH and cytotoxicity. Careful assessment and evaluation of the SAR and other desirable drug-like attributes of all synthesized molecules led to the identification of M029 (Figure 2a and Figure S2D, manuscript in preparation). To confirm the mode of action of M029, we also prepared its three structurally related but inactive analogs, PhClAc, M037, and M054 (Figure 2a). PhClAc represents the parent phenyl chloroacetamide warhead, which is inactive against SHP1 under the assay conditions. M037 is a negative control of M029 with a methoxy group in the central phenyl ring that negates SHP1 inhibitory activity. M054 loses the ability to inactivate SHP1 due to the removal of the chloride leaving group from the chloroacetamide warhead. Analysis of the *k*_obs_ versus inhibitor concentration revealed that, unlike Z57117023 (Figure 1c), the M029-mediated SHP1 inactivation displays saturation kinetics (Figure 2b), consistent with the two-step mechanism involving an initial reversible binding event before covalent adduct formation. This also suggests that M029 is associated with SHP1 with a higher affinity than that of Z57117023. The *k*_inact_ was determined to be 0.0038 ± 0.008 s^−1^, and the *K*_I_ was found to be 76.7 ± 12.5 μM at pH 7.0 and room temperature. Indeed, the *k*_inact_/*K*_i_ for SHP1 inhibition by M029 is 2973 M^−1^ min^−1^, which is 13-fold higher than that of Z57117023. Moreover, M029 is also 5-fold less reactive toward GSH than Z57117023, with a half-life of 34.3 h (Figure 2c). To determine the selectivity of M029 for SHP1, we measured the IC_50_ value for M029-mediated inhibition of a number of PTPs after a 60-min preincubation. As shown in Table 2, M029 exhibits an IC_50_ value of 2.6 μM for SHP1, and >22.4-fold selectivity over a large panel of mammalian PTPs, including its closely related homolog SHP2. To assess potential direct interactions between SHP1 and M029, we utilized the thermal shift assay to measure the thermal stability of SHP1 in the presence of M029. As shown in Figure 2d, M029, but not its inactive analog M037, caused a decrease of 1.8 °C in SHP1 protein melting temperature, indicating that M029 binding destabilizes SHP1 and supports SHP1 target engagement by M029. To corroborate the covalent interaction between SHP1 and M029, a single SHP1-M029 covalent adduct was observed by electrospray protein mass spectrometry when SHP1 was incubated with M029 (Figure 2e). The SHP1-M029 adduct formation resulted in a mass shift of 319.31 ± 1.14 Da (theoretical 321.01 Da), which corresponds to the molecular mass of M029 minus a chlorine atom. This is in line with the expectation that SHP1 inactivation by M029 occurs through nucleophilic substitution of the chlorine from the chloroacetamide warhead.

To further understand the mode of M029 action and ascertain whether M029 binds the SHP1 active site or a yet undefined novel site in SHP1, we performed a vanadate (VO_4_^3−^) protection experiment. Vanadate is a reversible and competitive PTP inhibitor,^[76,77]^ and thus excessive vanadate would be expected to fully occupy the PTP active site and prevent covalent modifications of residues within the active site pocket. In this assay, SHP1 was co-incubated with 30 μM M029 and 500 μM sodium orthovanadate or vehicle control for 30 min. The SHP1-inhibitor complex was then rapidly diluted 100-fold into a buffered solution containing 10 mM *p*NPP, and immediately, the absorbance at 405 nm was monitored over time to determine the remaining phosphatase activity. As expected, the SHP1•VO_4_^3−^ complex rapidly dissociated upon dilution, and SHP1 fully recovered activity compared to the vehicle-treated control (Figure 2f). Little residual phosphatase activity was detected when SHP1 was incubated with M029 alone, consistent with M029 irreversibly inactivating SHP1. We anticipated that covalent ligands exhibiting less or no SHP1 inhibition in the presence of vanadate could be considered as active site-directed. Interestingly, the jump-dilution experiment showed that SHP1 was not protected from M029 inactivation by the presence of vanadate (Figure 2f), indicating that M029 does not target the active site but likely engages with a non-active site SHP1 residue.

**Figure 3.**
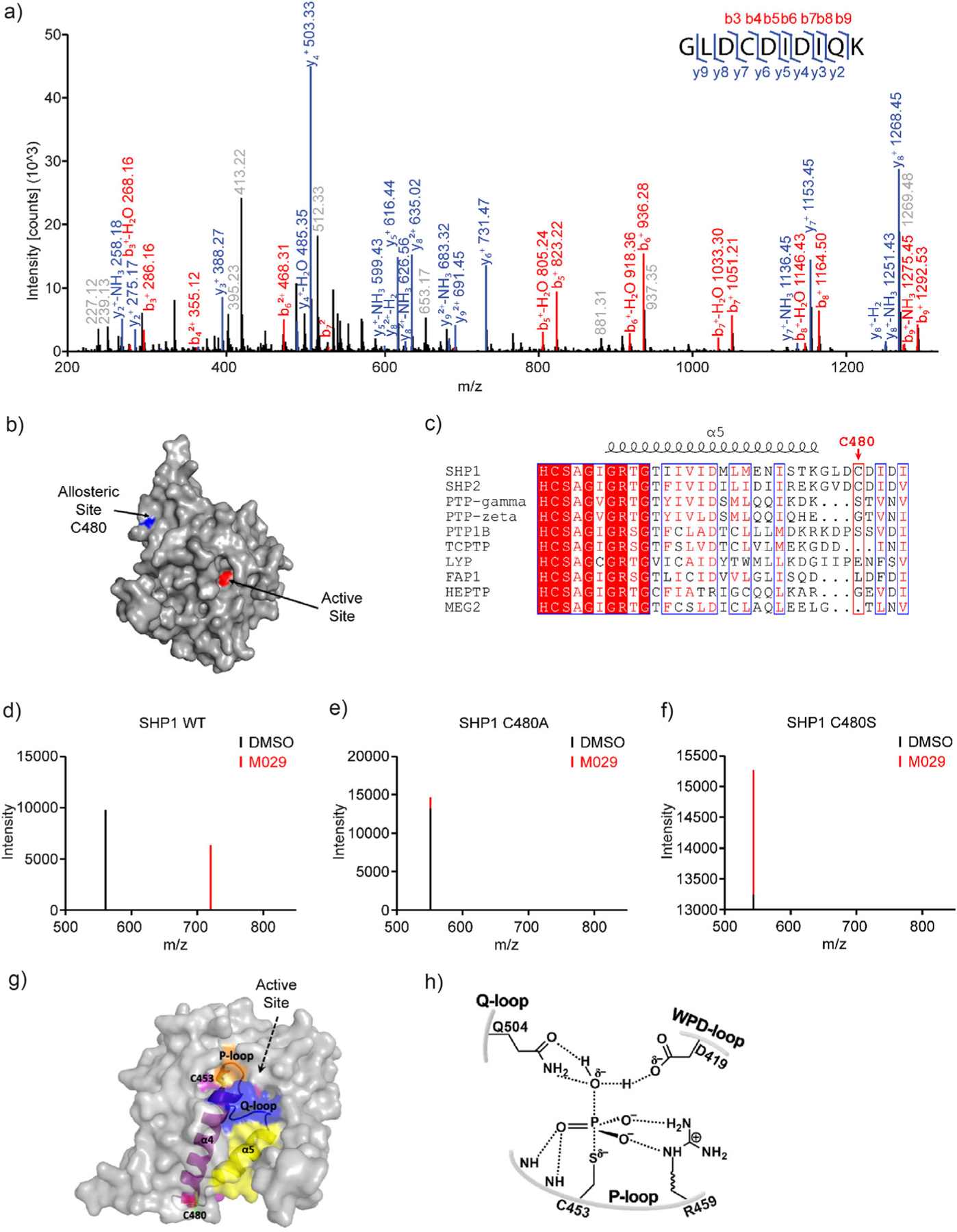
M029 is a covalent allosteric SHP1 inhibitor targeting a cryptic C480 unique to SHP1 and SHP2. a) SHP1 protein was incubated with or without M029 at room temperature for 60 min, and further MS/MS analysis revealed that M029 labeled the recombinant SHP1 protein at C480 on a peptide fragment GLDCDIDIQK. b) Space-filling model of SHP1 catalytic domain depicting the spatial relationship of the allosteric, cryptic C480 and the SHP1 active site. c) Amino acid sequence alignment of SHP1 with other PTPs surrounding Cys480. d–f) Cys480 was mutated to a structurally similar serine or alanine. Unlike SHP1 WT d), which showed C480 adduct at the peptide fragment GLDCDIDIQK, SHP1 C480A e) and SHP1 C480S f) did not show any M029 adduct formation at the corresponding peptide fragment GLDADIDIQK or GLDSDIDIQK, respectively, suggesting that M029 selectively modifies C480 in the GLDCDIDIQK peptide fragment. g) C480 (green stick and transparent surface colored by element) is buried under the protein surface and is linked with two catalytic loops (P-loop: orange cartoon; Q-loop blue cartoon) via two *α*-helices (labeled and shown in colored cartoon). The rest of the SHP1 structure is shown in transparent gray surface, and the active site is marked with a black arrow. The catalytic C453 is shown in stick and transparent surface (colored by element). h) SHP1 catalyzes the dephosphorylation of substrate proteins through the coordination of catalytic residues in the P-loop (Cys453 and Arg459), Q-loop (Gln504), and WPD-loop (Asp419); the perturbation of their precise positioning abolishes SHP1 enzymatic activity.

To ascertain where M029 binds to SHP1, the SHP1-M029 covalent adduct was proteolytically digested and analyzed by LC-MS/MS experiments. Mass spectrometry measurements revealed that M029 targets a single cysteine, Cys480 (Figure 3a), leaving several other cysteines in the protein, including the active site catalytic Cys453, untouched. Surprisingly, Cys480 resides in a cryptic site, which is opposite to the SHP1 active site (Figure 3b). Interestingly, Cys480 is unique to SHP1 and SHP2 and is not present in other members of the PTP family, partially accounting for the selectivity of M029 for SHP1 (Figure 3c). To further substantiate M029’s site specificity for SHP1 Cys480, we performed site-directed mutagenesis of Cys480 in order to determine its effect on SHP1-M029 adduct formation. Substitution of Cys480 by either an Ala or Ser completely eliminated SHP1-M029 adduct formation as evidenced by the disappearance of the peptide fragment harboring the M029-modified Cys480, signifying the Cys480 residue-selective modification by M029 (Figure 3d–f). To understand the mechanism by which M029 inactivates SHP1, we analyzed the crystal structure of the SHP1 catalytic domain^[78]^ and examined the spatial relationship between the cryptic Cys480 and SHP1 active site (Figure 3g). Cys480 is located between *α*4 and *α*5, which serve to position two active site loops, the P-loop and Q-loop, which harbor several active site residues and are known to be important for PTP catalysis (Figure 3h).^[53]^ We hypothesized that modification of Cys480 by M029 alters the conformation of these catalytically important loops and thereby repositions essential active site residues for substrate hydrolysis into a catalytically incompetent state. Consistent with this hypothesis, replacement of Cys480 with either an Ala or Ser significantly reduces *k*_cat_ (turnover number) without substantially affecting substrate binding (*K*_m_, Michaelis constant), which is indicative of an allosteric mode of inhibition (Table S1). Taken together, these results reveal that M029 is a covalent allosteric SHP1 inhibitor that inactivates SHP1 by specifically engaging Cys480, a cysteine residue within a cryptic and allosteric site away from the active site, through nucleophilic displacement of the Cl in the chloroacetamide moiety. These findings illustrate a new approach to selectively inhibit SHP1 by targeting a cryptic and allosteric site for inhibition.

### Cellular Target Engagement of SHP1 Covalent Allosteric Inhibitor M029

**Figure 4.**
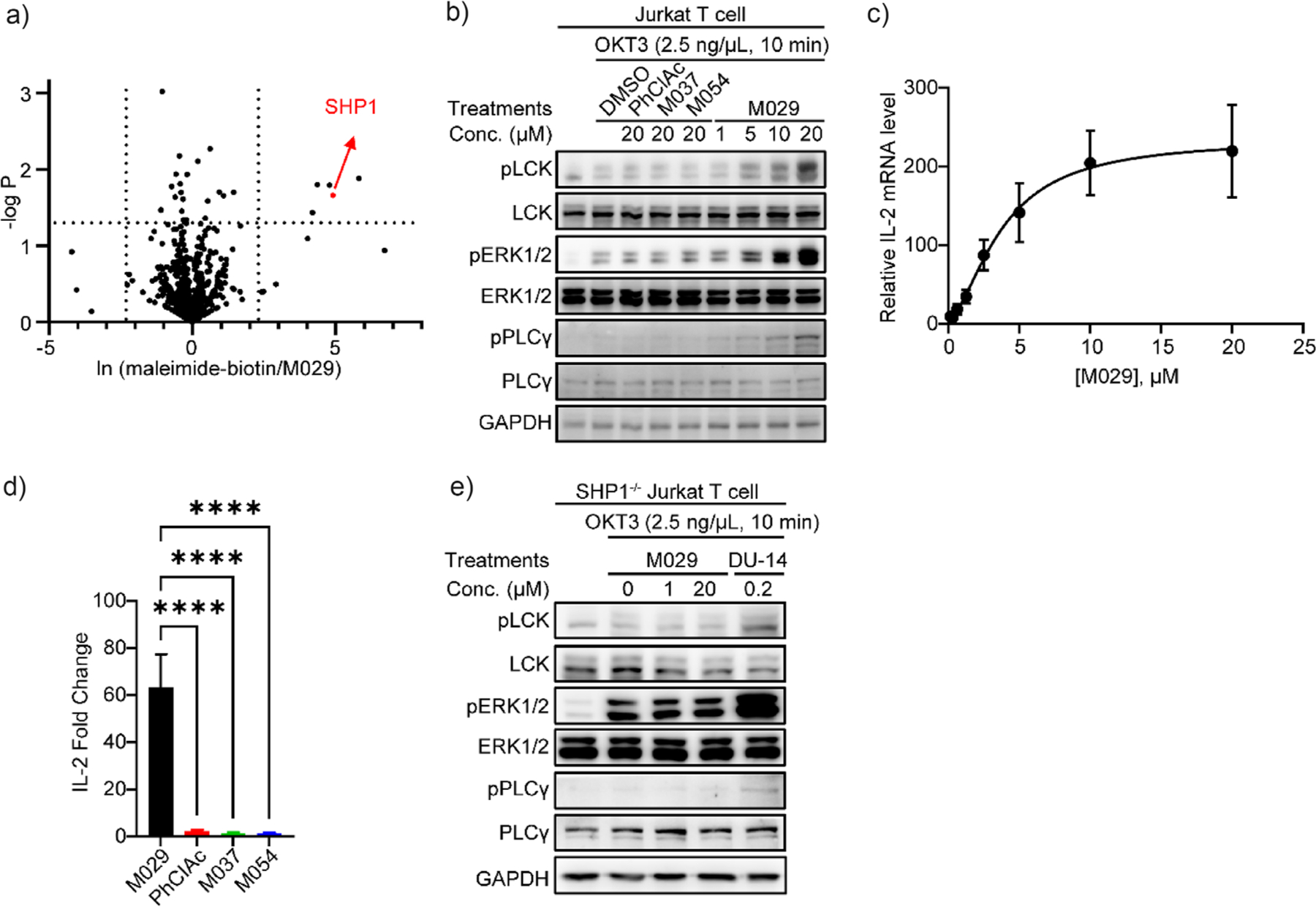
M029 engaged SHP1 in cellulo and showed strong efficacy in immune activation. a) Jurkat T cells were incubated with maleimide-biotin, a chemical probe that covalently modifies exposed cysteine residues in proteins, or M029. Supernatants from lysed cells were reduced, treated with iodoacetamide to cap thiol residues, and digested by trypsin for bottom-up proteomics analysis. The relative enrichment was calculated by ln (maleimide-biotin enrichment/M029 enrichment), where the cutoff of changes was set to 10-fold, and the cutoff of significance was set to the p-value of 0.05. b) Jurkat T cells were treated with M029 or negative controls, where only M029-treated cells showed an upregulation of LCK phosphorylation, ERK1/2 phosphorylation, and PLC*γ* phosphorylation in a dose-dependent manner. LCK is the direct substrate of SHP1 and ERK1/2, and PLC*γ* is the downstream effector protein of LCK-mediated TCR signaling. The enhanced phosphorylation of these proteins indicated the inhibition of SHP1 activity and accompanied TCR activation in cellulo. c) Jurkat T cells were treated with M029 in a dose-dependent manner to determine the relative IL-2 mRNA level, another biomarker for TCR activation, compared to the DMSO-treated groups. d) M029 increased relative IL-2 mRNA level while negative controls showed minimal effects even at 200 *μμ*, these results corroborated with the cell signaling and supported M029’s efficacy in TCR activation by SHP1 inhibition. Statistical analyses were performed with Graphpad Prism software 10.4.2 through the one-way ANOVA test using Turkey post-hoc comparison. ****p < 0.0001 was considered significant. Figures are plotted as average ± standard error of the mean (SEM). e) In SHP1 KO Jurkat T cells, the effect of M029 on LCK phosphorylation was abolished, while the control compound DU-14 (a dual degrader of PTP1B and TCPTP), which activates TCR through a SHP1-independent pathway, remains efficacious, confirming that M029 activates TCR signaling through increased LCK phosphorylation due to SHP1 inhibition.

Electrophilic fragments may engage unintended targets and exhibit potential off-target cytotoxicity, as often observed with covalent inhibition. To further define the selectivity of M029 for SHP1 and assess its cellular target engagement, a chemoproteomic investigation was performed to identify Cys residues protected by M029 from biotin-tagged maleimide from the whole proteome in Jurkat T cells.^[79]^ Since proteins labeled by M029 will be prevented from capture by the maleimide-biotin probe, M029-modified proteins can be readily identified by determining the ratio between the maleimide-biotin-bound proteins in the absence and presence of M029. Quantitative proteomic analyses revealed that out of the >2700 peptide fragments identified, SHP1 (Cys480) is the second most M029–labeled protein and one of only five proteins significantly blocked from maleimide modification by M029, with the other four being centrosomal or mitochondrial proteins (TSFM, NOA1, PCM1, and TIMM29) (Figure 4a). These observations showed that M029 effectively engages SHP1 inside the cells while displaying sufficient inert reactivity to minimize covalent attachment to other proteins. The modest observed off-target reactivity of M029 is not uncommon for targeted covalent inhibitors, including those FDA-approved covalent drugs targeting EGFR (Osimertinib),^[80,81]^ BTK (Ibrutinib),^[82]^ and KRAS (Sotorasib),^[83]^ which also modify other proteins in addition to the intended targets. To ascertain the potential off-target toxicity of M029, we treated Jurkat T cells, HEK293, MC38, EO771, B16-F10, and Raw264.7 cells with M029 and observed no significant cytotoxicity with up to 50 μM concentration (Figure S3). We speculate that the lack of cytotoxicity within the concentration range used might suggest that covalent modification of the four unintended proteins by M029 does not cause deleterious harm to the cell.

To further confirm M029 cellular target engagement, we investigated the effect of M029 on T cell function. SHP1 plays a negative role in T cells, and deletion of SHP1 in T cells lowers the activation threshold and enhances antitumor immunity.^[30–33]^ Mechanistically, SHP1 attenuates T cell receptor (TCR) signaling via dephosphorylation of the lymphocyte-specific protein tyrosine kinase LCK.^[31,84–86]^ T cell activation leads to increased expression and secretion of interleukin 2 (IL-2)^[87]^ while the removal of SHP1 in T cells enhances IL-2 production, cell proliferation, and cytolysis capacity.^[30,32]^ Accordingly, we sought to assess the impact of targeting SHP1 with M029 on TCR-LCK signaling and T cell activation. As expected, treatment of Jurkat T cells with M029 dose dependently increased the TCR-mediated phosphorylation of SHP1 substrate LCK as well as its downstream targets PLC*γ* and ERK1/2 (Figure 4b). M029 also dose-dependently promoted IL-2 production (Figure 4c). To confirm that the increases in pLCK, pPLC*γ*, and pERK1/2, as well as IL-2, were indeed caused by the M029-mediated SHP1 inhibition, we also evaluated the effect on Jurkat T cell signaling by three structurally related but inactive analogs of M029, PhClAc, M037, and M054 (Figure 2a). As anticipated, these negative control compounds neither impacted the phosphorylation levels of LCK, PLC*γ*, and ERK1/2 nor the IL-2 secretion even at 20 μM concentration (Figure 4b,d). To further substantiate M029 target engagement, we found that M029 was unable to alter LCK, PLC*γ*, and ERK1/2 phosphorylation when SHP1 was deleted from Jurkat T cells (Figure 4e). In contrast, DU-14, a small molecule degrader of PTP1B and TC-PTP, both of which also negatively regulate TCR signaling,^[88]^ could still enhance the TCR-induced LCK pathway activation in SHP1^−/−^ Jurkat T cells (Figure 4e). These findings corroborate that the observed increase in LCK phosphorylation and its downstream signaling indeed resulted from SHP1 inhibition by M029. Taken together, our data demonstrates that M029 is a genuine covalent allosteric SHP1 inhibitor that possesses an excellent balance between its tempered intrinsic reactivity and near exquisite selectivity for SHP1, supporting its high degree of target engagement inside the cell. Consistent with genetic and biochemical studies,^[30–33,84–86]^ the results also demonstrate that SHP1 inhibition by M029 can enhance TCR-LCK signaling and T cell activation, which are important for antitumor immunity.

### M029 is Orally Bioavailable and Suppresses Syngeneic Tumor Growth in Immunocompetent Mice

Given M029’s superb potency, selectivity, and target engagement, we considered that these desirable attributes make it a powerful chemical probe for interrogating the roles of SHP1 in biological processes and a promising lead for further development into an SHP1-targeted therapy. Previous studies showed that genetic deletion of SHP1 promotes antitumor immunity in syngeneic tumor models, including MC38 and EO771.^[40]^ To demonstrate the translatability of pharmacological targeting of SHP1 for novel immunotherapy, we sought to investigate whether systemic SHP1 inhibition with M029 is feasible and phenocopies the genetic knockout. To that end, we determined the pharmacokinetic (PK) properties of M029 in mice by administrating the compound via intraperitoneal (IP), intravenous (IV), and oral (PO) routes at a concentration of 25 mg/kg (Figure S4). Thus, dosing M029 intraperitoneally at 25 mg/kg yielded a compound half-life (t_1/2_) of 36.3 ± 5.7 min, a peak plasma concentration (C_max_) of 122.1 ± 28.2 μM, and an area under the curve (AUC) of 5,094 ± 575.5 μM·h. Importantly, M029 is orally bioavailable. Indeed, oral administration of M029 at 25 mg/kg furnished a t_1/2_ of 38.5 ± 7.6 min, C_max_ of 29.8 ± 7.1 μM, and an AUC of 1,411 ± 221.2 μM·h, with an of oral bioavailability (%F) 18%.^[89]^ Interestingly, the half-lives of M029 are comparable to those of BTK and KRAS covalent drugs used in the clinic.^[51,90]^ Moreover, even with the 25 mg/kg oral gavage, the plasma concentration of M029 was maintained several-fold higher than its biochemical IC_50_ and two-fold above its cellular EC_50_ for Jurkat T cell activation for at least 1 h duration (Figure S4). These PK properties ensure that adequate M029 compound exposure can be achieved to assess the effect of SHP1 inhibition in vivo.

**Figure 5.**
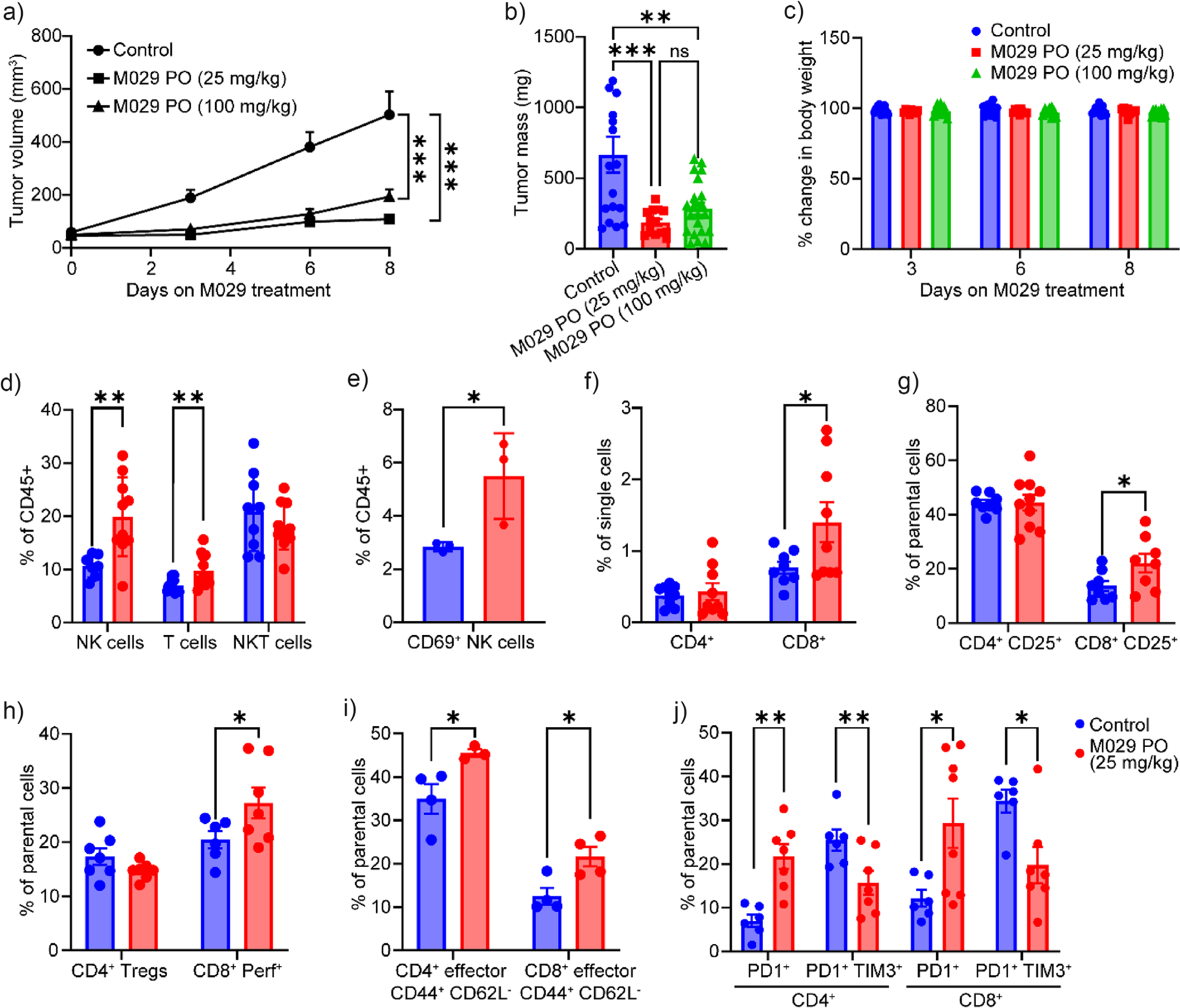
M029 inhibits syngeneic MC38 tumor growth by promoting infiltration and activation of both T cells and NK cells in the tumor microenvironment. Oral dosing of mice with 25 mg/kg (*n* = 11) or 100 mg/kg (*n* = 12) of M029 significantly delayed the MC38 tumor progression compared to the control group treated with vehicle (*n* = 10). Both a) tumor volume and b) tumor weight showed significant decreases upon M029 treatment, while c) the body weight of all groups showed no significant changes, suggesting that M029 is efficacious and of low acute toxicity in vivo. The tumor tissues from each group of mice were isolated and examined through flow cytometry. d) Compared to the control group, mice treated with 25 mg/kg M029 showed significantly higher NK cells and T cells in the tumor microenvironment. e) Among the infiltrated NK cells, a higher CD69^+^ population was observed in the M029-treated group, suggesting enhanced NK activation. In the meantime, f) more CD8^+^ T cells were observed in the infiltrated T cell population, which are the major tumor cell-killing cells. The infiltrated CD8^+^ T cells showed higher g) CD25^+^ population and h) perforin^+^ population, which are the activation biomarker and a cytotoxicity biomarker, respectively, suggesting that the infiltrated CD8^+^ T cells are more activated than the control group, consistent with M029’s mode of action. This T cell activation was further supported by i) the increased effector CD8^+^ population in the tumor microenvironment. Additionally, j) M029 treatment also significantly reduced the expression of PD1^+^TIM3^+^ on both CD4^+^ and CD8^+^ T cells, which suggested that fewer T cells are going into exhaustion, corroborating the strong antitumor efficacy observed from M029. Statistical analyses were performed with Graphpad Prism software 10.4.2 through the student t test or the one-way ANOVA test using Turkey post-hoc comparison. *p < 0.05, **p < 0.01, ***p < 0.001, and ****p < 0.0001 were considered significant. Figures are plotted as average ± standard error of the mean (SEM).

Given its favorable PK profiles, we evaluated the antitumor activity of M029 in C57BL/6 mice bearing MC38 colorectal tumors. Mice bearing established tumors were given once daily with either saline, 25 mg/kg (IP), 25 mg/kg (PO), or 100 mg/kg (PO) M029. In line with the phenotypes observed with SHP1 deletion, M029 treatment, either by oral gavage at 25 or 100 mg/kg or IP injection at 25 mg/kg, effectively halted tumor progression in mice bearing syngeneic MC38 tumors (Figure 5a,b and Figure S5A,B). The compound was well tolerated in mice, since there were no obvious signs of toxicity observed even when M029 was dosed at the higher concentration of 100 mg/kg, as evidenced by the lack of adverse effects on body weight and organ histology (Figure 5c and Figure S5C,D). These findings demonstrated that SHP1 is a druggable target for immunotherapy and systemic SHP1 inhibition with M029 confers antitumor activity with no observable side effects, consistent with M029’s inherent low chemical reactivity and exceptional target selectivity.

To elucidate the mechanism by which M029 elicits its antitumor activity, we analyzed the immune cell population within the tumor samples. Profiling of MC38 tumor immune infiltrates revealed a ~33% and ~100% increase of CD3^+^ T and NK1.1^+^ NK cells, respectively, but not the NKT cells in M029-treated tumors (Figure 5d). In addition, the tumor-infiltrating NK cells were significantly more activated in M029-treated mice as indicated by the increased presence of the activation marker CD69 (Figure 5e). Moreover, M029 treatment also led to an increased infiltration of cytotoxic CD8^+^ T cells but not the CD4^+^ T cell population (Figure 5f). Functional marker profiles within the infiltrating CD8^+^ T cells showed that M029 enhanced T cell activation, cytotoxicity, and effector function as evidenced by the higher expression of activation marker CD25 (Figure 5g), cytotoxicity marker perforin (Figure 5h), and effector markers CD44^+^CD62L^−^ (Figure 5i). These findings indicate that M029 inhibits tumor progression by promoting the infiltration and activation of NK and T cells in the tumor, which are consistent with previous genetic studies demonstrating that SHP1-deficient NK cells and T cells exhibit enhanced antitumor activity.^[34,35,37–39]^ Interestingly, although the number of pre-exhausted PD1^+^TIM3^−^ T cells increased, we observed a decline in the population of late exhaustion T cells (PD1^+^TIM3^+^) in mice treated with M029, which suggests M029 impairs the progression of T-cell exhaustion to sustain CD8^+^ T-cell antitumor activity (Figure 5j). This observation is in line with earlier reports that SHP1 activity contributes to LAIR1-mediated T cell exhaustion.^[91]^

**Figure 6.**
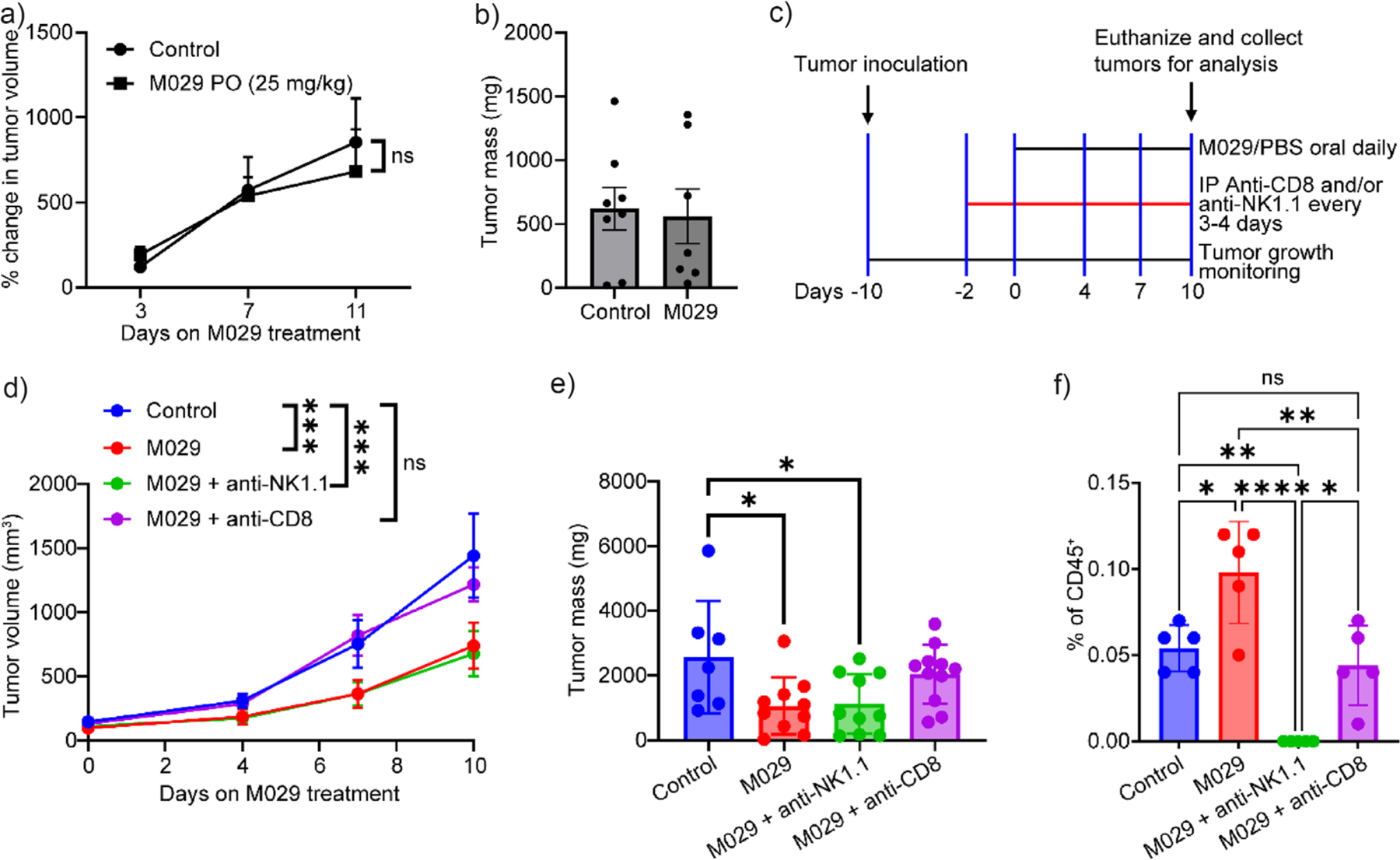
Cytotoxic T-cell activity is necessary for M029 antitumor activity. Nude mice lacking the adaptive immune system bearing syngeneic MC38 models were treated with either 25 mg/kg M029 (*n* = 4) or control vehicle (*n* = 4), where no significant a) tumor volume change or b) tumor weight change was observed, indicating that the adaptive immune system is critical for M029’s antitumor efficacy. c) Workflow for CD8^+^ and NK cell depletion studies. C57BL/6 mice were injected with anti-CD8 or anti-NK1.1 antibodies every 3–4 days for 8 days after tumor inoculation. Mice were orally dosed with M029 or control vehicle every day for 10 days after tumor inoculation at the same time (Control, *n* = 4; M029, *n* = 6; M029 + anti-NK1.1, *n* = 6; M029 + anti-CD8, *n* = 6). NK cell depletion did not impact M029’s anticancer efficacy as demonstrated by the d) tumor volume change and e) tumor weight change, while the CD8^+^ T cells depletion completely abolished the M029 antitumor efficacy as demonstrated by the comparable tumor volume and weight changes to the control group. f) The tumor tissues from each group of mice were isolated, and the NK cell population was examined by flow cytometry. The NK cell depletion was successful, as marked by the absence of NK cells in the anti-NK1.1 group, while the NK cell population in the anti-CD8 treatment group was lower than M029-only treatment and comparable to the vehicle control group. This difference implied that the CD8^+^ T cells were critical to induce the NK cell infiltration in M029-treated mice, and the depletion of CD8^+^ T cells diminished M029’s efficacy because of the absence of activated T cells and infiltrated NK cells. Statistical analyses were performed with Graphpad Prism software 10.4.2 through the student t test or the one-way ANOVA test using Turkey post-hoc comparison. *p < 0.05, **p < 0.01, ***p < 0.001, and ****p < 0.0001 were considered significant. Figures are plotted as average ± standard error of the mean (SEM).

To further establish whether the inhibitory effects of M029 on tumor progression could be attributable to enhanced antitumor immune responses, we treated MC38 tumors in nude mice. Conforming to the known function of SHP1 in immune cells, M029 was ineffective in inhibiting tumor growth in nude mice (Figure 6a,b), which underscores the necessity of a fully functional immune system for M029 to exert its antitumor activity. This finding also indicates that the M029-mediated protective effects against tumor growth were immune-specific. To evaluate the importance of CD8^+^ T and NK cells in M029-induced antitumor activity, we induced CD8^+^ T cell or NK cell depletion in the MC38 syngeneic model using anti-CD8 or anti-NK1.1 antibodies as described in Figure 6c. We confirmed the depletion of CD8^+^ and NK cells in various treatment groups using flow cytometry, as shown in Figure S6. Notably, M029 exhibited no antitumor efficacy in mice with CD8^+^ T-cell-depleted (Figure 6d,e), emphasizing the importance of cytotoxic T-cell activity for effective tumor growth inhibition by M029. In contrast, the antitumor effects of M029 remained unaffected in NK cell-depleted mice (Figure 6d,e). Taken together, our findings showed that CD8^+^ T cell deficiency completely negated the in vivo efficacy of M029 and suggested that M029’s antitumor activity is predominantly through the activation of the T cells. These findings are consistent with the known role of SHP1 in T cells and with our in vitro data that indicate M029 enhances T cell activation with no direct effect on tumor cells. Because NK cell depletion did not significantly compromise the efficacy of M029, we conclude that the M029 effect in the MC38 syngeneic model is less dependent on NK cells. Additionally, we found that CD8^+^ T cell depletion led to the abolishment of the increase in NK cell tumor infiltration induced by M029 (Figure 6f), which suggests that the NK cell infiltration is enhanced by increased CD8^+^ T cell activity. These findings align with previous reports that activated CD8^+^ T cells recruit NK cells to tumors through releasing cytokines and chemokines like IFN-*γ*.^[92]^ The observed changes in immune activity induced by M029, such as increased tumor infiltration of cytotoxic CD8^+^ T cells, enhanced T cell cytotoxicity and effector function, as well as improved NK cell infiltration and activation, collectively contribute to the anticancer effects exerted by M029 treatment.

## Conclusion

Contemporary immunotherapies have transformed cancer treatment, yet many patients still fail to respond to the prevailing options. Moreover, since the majority of the current approaches target surface immune checkpoint receptors with large biologics, they have limited tissue penetration to the tumor microenvironment. Hence, there remains a clear need for better solutions and continued innovation through both basic and translational research. To that end, the pursuit of small molecules targeting intracellular regulatory mechanisms has emerged as an increasingly attractive avenue for novel immunotherapy. SHP1 has recently emerged as an intriguing therapeutic target for small-molecule immunotherapeutic agents, because it is an important intracellular mediator of inhibitory signals in the immune system, and deletion of SHP1 promotes antitumor immunity in mice.^[18–20]^ Thus, SHP1 inhibition is expected to enhance the tumoricidal capacity of immune cells and boost host antitumor immunity. However, despite its known implications for cancer immunotherapy, SHP1 remains unexplored as a drug target due to the absence of high-quality small-molecule inhibitors. Indeed, drug discovery targeting PTPs remains a major challenge, as these enzymes possess a conserved and polar active site not readily addressed by conventional strategies.^[42–44]^ Here, we report our effort in developing covalent SHP1 inhibitors, as targeted covalent inhibition has risen as a powerful solution for accessing protein sites in difficult-to-drug targets.^[45–50]^ In addition, covalent inhibitors form irreversible bonds with specific residues on target proteins, leading to sustained inhibition even after compound clearance, and have the potential to display increased selectivity within homologous proteins by modifying non-conserved residues. One key feature for the successful development of targeted covalent inhibitors into therapeutics, however, requires balancing the electrophile’s intrinsic chemical reactivity that enables selective target engagement while also reducing its cross-reactivity with undesired proteins or other endogenous nucleophiles such as glutathione. The goal of this study is to develop SHP1 covalent inhibitors with the requisite potency, selectivity, tempered reactivity, and drug-like properties to interrogate the therapeutic potential of SHP1 as a cancer immunotherapy target.

We applied a high-throughput screening approach using a library of covalent fragments and discovered a phenyl chloroacetamide-based molecule, Z57117023, that can irreversibly inhibit SHP1 phosphatase activity. By carefully exploring the structure–activity relationship and carrying out a multiparameter optimization of Z57117023, we developed a first-in-class orally efficacious covalent allosteric SHP1 inhibitor M029 with greatly attenuated chemical reactivity, increased affinity and selectivity for SHP1, and excellent drug-like properties. Mass spectrometry and site-directed mutagenesis experiments revealed that M029 inhibits SHP1 activity by covalently modifying a non-conserved Cys480 that is unique to SHP1 and SHP2 among the PTPs. Importantly, Cys480 resides in a cryptic site opposite to the highly conserved PTP active site, underscoring a novel allosteric mechanism for M029-mediated SHP1 inhibition, likely through perturbation of the active site structure or stabilization of a catalytically inactive conformation.^[93]^ Although covalent modification of both PTP active site and non-active site Cys residues has previously been described,^[54–61,94,95]^ Cys480 has never been covalently targeted. Thus, optimization of an electrophile fragment that reacts with cysteines led to the discovery of a cryptic ligandable Cys480 in SHP1 that was not previously known to be druggable. These findings illustrate that targeting a cryptic Cys residue remote from the catalytic site can be exploited in allosterically inhibiting SHP1, which circumvents the challenges posed by the chemistry of the enzyme active site. Moreover, these results suggest a new strategy for inhibiting a promising immunotherapy target.

In line with the desirable attributes expected from both covalency and allostery,^[96]^ the covalent allosteric inhibitor M029 irreversibly inactivated SHP1 with outstanding selectivity. Indeed, despite carrying a chloroacetamide warhead, M029 engages only SHP1 and 4 other unrelated proteins in the whole cell proteome. Among the 4 additional M029 labeled proteins, TSFM (Mitochondrial Translation Elongation Factor Ts), NOA1 (Nitric Oxide Associated 1), and TIMM29 (Translocase of Inner Mitochondrial Membrane 29) are all functionally linked to mitochondrial translation, protein import, and respiratory homeostasis, while PCM1 (Pericentriolar Material 1) regulates centrosome integrity and mitotic fidelity. Covalent modification of these proteins by M029 in cells could induce mitochondrial dysfunction and promote mitotic errors and chromosomal instability.^[97,98]^ However, M029 displays no obvious cytotoxicity within the concentration range used in a number of cell lines, suggesting that M029 is a “silent ligand” for these proteins and does not perturb their physiological functions. Notably, M029 can specifically block SHP1-dependent signaling inside the cell. In particular, inhibition of SHP1 by M029 augmented the TCR-mediated phosphorylation of LCK and its downstream targets PLC*γ* and ERK1/2 in T cells, leading to T cell activation as evidenced by the increased cytokine production (e.g., IL-2). Importantly, M029 exhibits excellent pharmacokinetic properties and is orally bioavailable. Most significantly, SHP1 inhibition by M029 phenocopies the antitumor effects seen in genotypic SHP1 knockout mice.^[40]^ Thus, M029 inhibits syngeneic tumor growth and demonstrates robust intratumor target engagement as evidenced by the increased infiltration of NK cells and CD8^+^ T cells, improvements in their effector functions, and cytotoxicity of the infiltrated CD8^+^ T cells. Moreover, although increased T cell exhaustion is commonly encountered in immunotherapies, M029 treatment caused a delay in the exhaustion process of T cells, which may contribute to its immunotherapeutic efficacy. Together, these studies provide direct pharmacologic validation of SHP1 as a promising immunotherapy target and highlight the translational potential of systemic targeting of SHP1 as a novel therapeutic approach for cancer treatment.

In summary, we showed that covalent fragment screening can be used to rapidly identify highly potent and selective electrophiles targeting the PTPs. We developed an orally active, covalent allosteric SHP1 inhibitor M029, which engages Cys480, a cysteine residue within a novel cryptic and allosteric site in SHP1. This work expands the range of PTP cysteines that can be targeted for potent and selective allosteric control of PTP activity^[96]^ and offers an auspicious avenue in circumventing the difficulties that have limited the development of active site-directed PTP inhibitors. We also demonstrated that this first-in-class covalent allosteric SHP1 inhibitor is efficacious as a monotherapy in a syngeneic MC38 mouse cancer model by triggering robust antitumor immunity, enhancing NK and CD8^+^ T cell function, and reducing T cell exhaustion. Collectively, this study establishes that small molecule SHP1 inhibition is a compelling approach for new cancer immunotherapy and provides a prototype lead compound that can be further optimized for eventual clinical applications.

## Supplementary Material

SI

Supporting Information

All the in vivo studies were performed under an animal protocol (1511001324) approved by the Institutional Animal Care & Use Committee of Purdue University, in accordance with the recommendations in the Guide for the Care and Use of Laboratory Animals of the National Institutes of Health.

Additional supporting information can be found online in the Supporting Information section

## Figures and Tables

**Table 1:** IC_50_ values after 10-min or 60-min preincubation between compounds and proteins of 11 confirmed hits with selectivity for SHP1 over SHP2, PTPN22, and PTP1B.^a)^

	SHP1		SHP2		PTPN22		PTP1B	
Confirmed hits	10 min	60 min	10 min	60 min	10 min	60 min	60 min	10 min
Z7154	38.0	8.0	>>200	47.1	3710	112.0	>>200	37.1
Z8803	46.1	10.0	>>200	81.06	745.3	100.8	>>200	140.6
Z0188	101.5	23.0	>>200	125.0	>>200	>> 200	>>200	122.6
Z1943	59.4	15.0	>>200	>> 200	>>200	>> 200	>>200	>> 200
Z57117023	99.1	17.5	>>200	>> 200	>>200	>> 200	>>200	>> 200
Z2050	57.9	10.3	>>200	>> 200	>>200	>> 200	>>200	>> 200
Z4666	196.0	38.8	>>200	>> 200	>>200	>> 200	>>200	>> 200
Z7520	>>200	56.8	>>200	>> 200	>>200	>> 200	>>200	>> 200
Z0193	190.8	35.3	>>200	97.7	900.2	65.8	>>200	>> 200
Z0590	56.8	21.2	>>200	147.9	>>200	>> 200	>>200	>> 200
Z1771	172.7	33.7	>>200	>> 200	>>200	>> 200	>>200	>> 200

a) IC_50_ values are in μM.

**Table 2:** Selectivity of M029 against a large panel of PTPs.

PTP	60 min IC_50_ (μM)
SHP1	2.6 ± 0.5
SHP2	58.2 ±4.6
PTP1B	152.3 ± 16.6
PTPN22	88.6 ± 20.1
FAP1	>>200
PEST	>>200
PEZ	172.1 ± 19.3
CDC14A	>>200
MKP5	165.2 ± 20.1
PP5	>>200
STEP	>>200
Ssu72	>>200
HePTP	>>200
LMPTP	>>200
TCPTP	149.9 ± 31.1
CD45	139.3 ± 38.5
VHR	179.8 ± 46.8

## Data Availability

The data that support the findings of this study are available in the supplementary material of this article.
