# Supplementary material for: Discovery of a First‐in‐Class Covalent Allosteric SHP1 Inhibitor with Immunotherapeutic Activity
Source: Angew Chem Int Ed Engl. 2025 Dec 26;65(7):e25126. doi: 10.1002/anie.202525126 (PMC12833730; doi:10.1002/anie.202525126)
Supplement: Supplementary file 1 — Supporting Information [file ANIE-65-e25126-s001.docx]

**Supporting Information**

**Discovery of a first-in-class covalent allosteric SHP1 inhibitor with immunotherapeutic activity**

Zihan Qu^a#^, Frederick Nguele Meke^b#^, Zheng Zhang^c^, Aaron D. Krabill^b^, Christine S. Muli^b^, Brenson A. Jassim^b^, Jiajun Dong^b^, Quyen D. Nguyen^a^, Yunpeng Bai^b^, Jinyue Li^a^, Yiming Miao^b^, Bardia Asadi^b^, Levi M. Johnson^a^, Jinmin Miao^b^, Darci J. Trader^b^, W. Andy Tao^a,c,d,e^, and Zhong-Yin Zhang^a,b,d,e^*

^a^James Tarpo Jr. and Margaret Tarpo Department of Chemistry, ^b^Borch Department of Medicinal Chemistry and Molecular Pharmacology, ^c^Department of Biochemistry, ^d^Institute for Cancer Research, and ^e^Institute for Drug Discovery, Purdue University, 720 Clinic Drive, West Lafayette, IN 47907, USA

^#^Z.Q. and F.N.M. contributed equally to this work.

^*^To whom correspondence should be addressed. Email: [zhang-zy@purdue.edu](mailto:zhang-zy@purdue.edu)

Table of Contents

1. Supplemental Figures and Tables P3
2. Experimental Procedures P10
3. References P24
4. NMR Spectra P25

Supplemental Figures and Tables

**
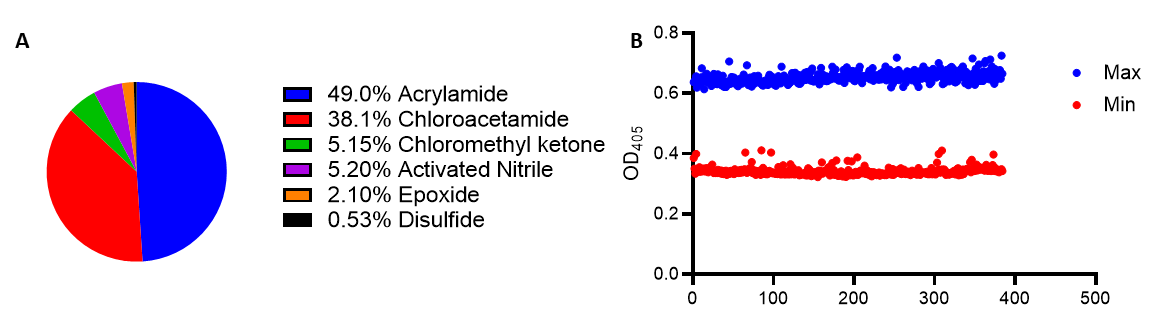
**

**Figure S1**. Chemical composition and screening assay validation for SHP1 HTS. (A) Component of the electrophile focus library assembled. (B) Z’ of SHP1 screening assay was determined to be 0.70 from 384 maximal signals (SHP1 and pNPP) and 384 minimal signals (substrate only).


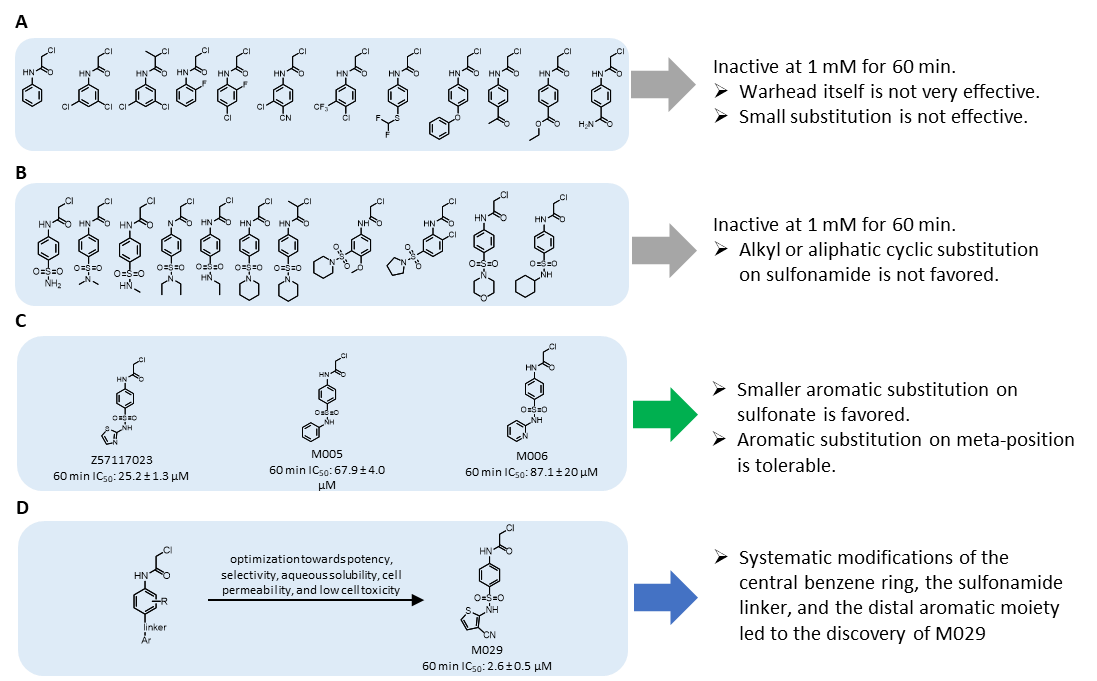
**Figure S2**. SAR by catalog and subsequent optimization led to the discovery of M029. (A) Phenyl chloroacetamides with or without small/medium substitutions were inactive, suggesting the importance of the linker and terminal ring structure. (B) Saturated substitutions of the sulfonamide linker rendered compounds ineffective, suggesting the importance of the aromaticity of the terminal ring on compound potency. (C) Aniline and pyridine substitutions on the linker showed lower potency than thiazole substitution, implying the preference for compact scaffolds. (D) Further systematic optimization of the SHP1 inhibitors led to the discovery of M029. A series of compounds featuring diverse substitutions on the central benzene ring, various linkers, and different distal aromatic moieties were synthesized and evaluated for in vitro SHP1 inhibition and selectivity over other PTPs. The most promising candidates were further characterized for key medicinal chemistry liabilities, including aqueous solubility, membrane permeability, glutathione reactivity, and cytotoxicity. Based on a favorable balance of SHP1 potency, selectivity, physicochemical, and ADME-relevant properties, M029 was advanced as the lead compound for subsequent studies.

**Figure S3**. Cancerous cells (MC38, EO771, and B16-F10), non-cancerous cells (HEK293), and immune cells (Jurkat and Raw264.7) were treated with M029 at different doses for 24 hours, and the cell viability was determined by the CCK8 experiments using DMSO-treated cells as a control. No significant toxicity was observed with up to 50 μM M029, which is well above the working concentration of M029 in cells and *in vivo*. Results were analyzed using GraphPad Prism 10.4.2. The figure is plotted as average ± standard error of the mean (SEM).

**
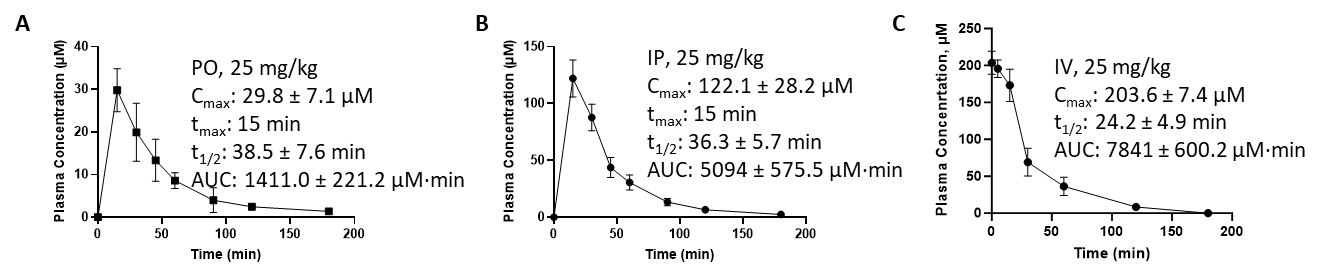
**

**Figure S4**. Pharmacokinetic parameters of M029. Curves of (A) PO, (B) IP, (C) IV dosage of M029 at 25 mg/kg in WT C57BL/6 mice. Results were analyzed using GraphPad Prism 10.4.2. The figure is plotted as average ± standard error of the mean (SEM).


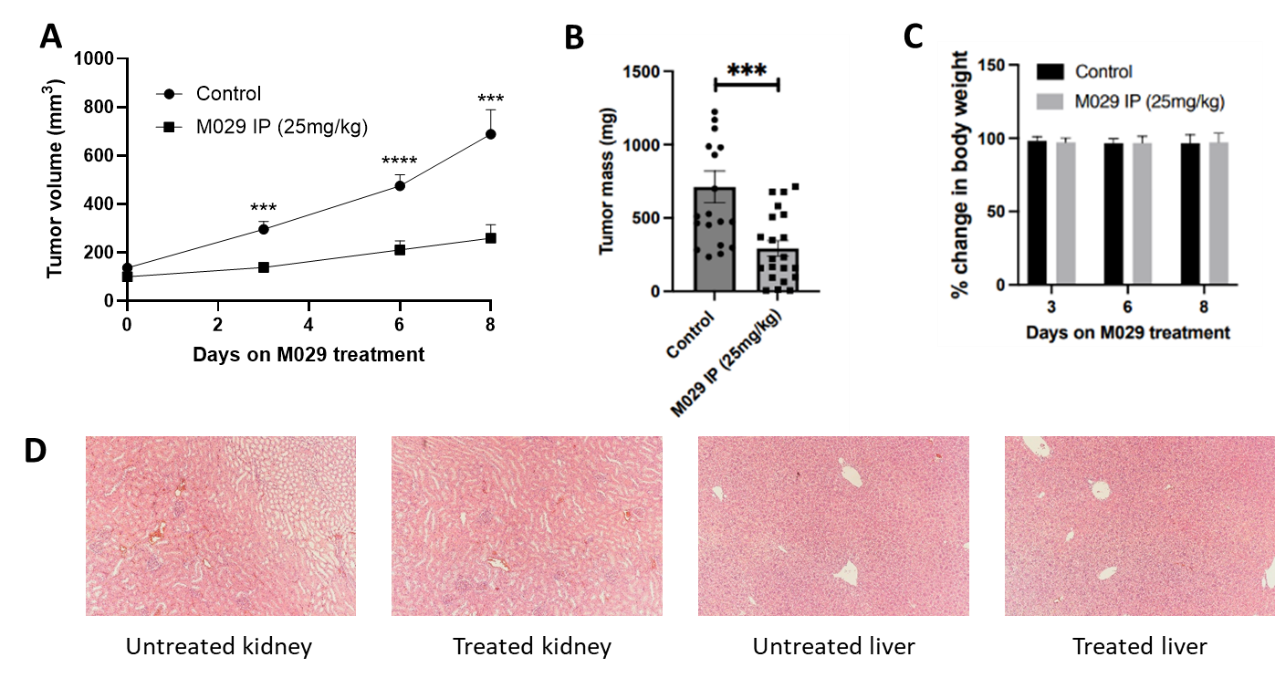


**Figure S5**. IP dosage of M029 delayed MC38 tumor progression without acute toxicity. (A) Tumor volume progress and (B) Final tumor weight comparison of the MC38 tumor with IP dosage of 15 mg/kg M029 (n=11) or vehicle control (n=11). (C) Body weight comparison of M029 throughout the study. (D) Tissue section of kidneys and livers from M029 or vehicle-treated mice. No significant lesion or tissue changes were observed for M029-treated mice. Statistical analyses were performed with Graphpad Prism software 10.4.2 through the student t test or the one-way ANOVA test using Turkey post-hoc comparison. *p<0.05, **p<0.01, ***p<0.001, and ****p<0.0001 were considered significant. Figures are plotted as average ± standard error of the mean (SEM).

**
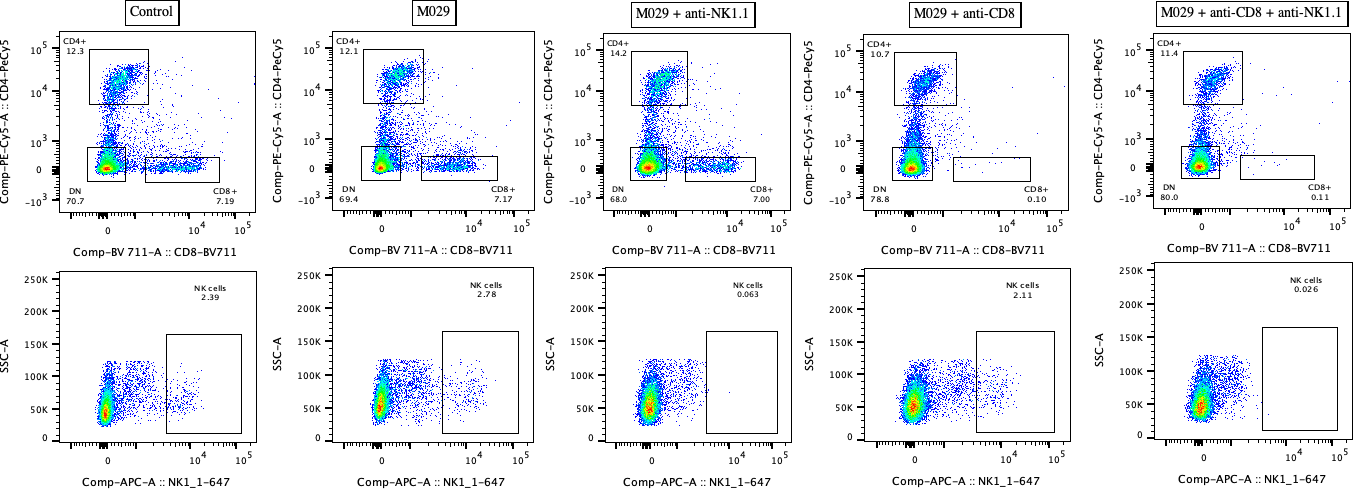
**

**Figure S6**. Representative flow cytometry results of CD8^+^ T/NK cell depletion studies. In the control group and the M029-treated group, CD8^+^ T cells make up ~7% of immune cells and NK cells make up ~2% of immune cells. For mice treated with anti-NK1.1 antibody, the NK cell population is negligible (<0.1%). For mice treated with anti-CD8^+^ antibody, the CD8^+^ T cell population dropped to ~0.1%. Mice treated with both antibodies showed the absence of both CD8^+^ T cells and NK cells. The CD4^+^ T cells were used as a benchmark to ensure the entire immune cell population was not significantly impacted by antibody treatment.

**Table S1.** Comparison of enzyme kinetics of wild-type SHP1 against SHP1 C480A and SHP1 C480S mutants

|  | ***k*_cat_ (s^-1^)** | ***K*_m_ (mM)** | ***k*_cat_/*K*_m_ (mM^-1^ s^-1^)** |
| --- | --- | --- | --- |
| **WT** | 18.4 ± 2.6 | 4.3 ± 0.3 | 4.3 ± 0.2 |
| **C480A** | 7.0 ± 1.6 | 3.9 ± 0.4 | 1.8 ± 0.2 |
| **C480S** | 8.1 ± 0.5 | 5.5 ± 1.8 | 1.5 ± 0.3 |

Experimental Procedures

**Materials and Reagents**

Unless otherwise noted, all reagents were purchased from commercial suppliers and used without further purification. The cysteine-focused electrophile fragment library was handpicked from Enamine. Thin-layer chromatography was performed using glass precoated Merck silica gel 60 F254 plates. Column chromatography was performed using KP-SIL silica gel (Biotage, USA), and flash column chromatography was performed on Biotage prepacked columns using the automated flash chromatography system Biotage Isolera One. HPLC purification was performed using column: Phenomenex Kinetex C18 5 μm 150 x 21.2 mm; Eluent A: water + 0.1% formic acid (99%), Eluent B: methanol; DAD scan: 210-400 nm. The 1H- and 13C- NMR spectra were recorded on a Bruker AVANCE 500 MHz spectrometer using dimethyl sulfoxide (DMSO-d6) as the solvent. Chemical shifts are expressed in ppm (δ scale) and referenced to the residual protonated solvent. Peak multiplicities are reported using the following abbreviations: s (singlet), d (doublet), t (triplet), q (quartet), m (multiplet), or br (broad singlet). Mass spectra and purity data were obtained using an Agilent Technologies 6470 series, triple quadrupole LC–MS. The purity of all final tested compounds was determined to be >95% (UV, λ = 254 nm). High-resolution mass analysis was performed on an Agilent 6550 iFunnel Q-TOF mass LC–MS. pNPP was purchased from Thermo Scientific (catalog#PI34045).

**Cloning, expression, and purification of PTP proteins for enzymatic assays**

cDNAs encoding recombinant PTPs (TC-PTP, residues 1-387; PTP1B, residues 1-321; SHP1, residues 245-543; SHP2, residues 224-528; LYP/PTPN22, residues 1-294; STEP, residues 258-539; HePTP, residues 22-360; PTP-PEST, residues 5-304; FAP1, residues 2124-2485; PTPα, residues 173-793; PTPε, residues 107-697; CD45, residues 620-1236; CDC14A, residues 1-413; CDC14B, residues 1-411; MKP5, residues 320-647; VHZ, residues 1-150; Laforin, residues 1-331; LMWPTP, residues 1-158) were amplified by PCR and subcloned into the pET28a(+) bacterial expression vector to allow for the expression of N-terminal His-tagged proteins. E. coli BL21(DE3) (Novagen) was used as an expression host, and the induction of protein expression was carried out in LB Broth with 1 mM IPTG at 18 °C overnight. Cell pellets were stored at −80 °C for subsequent protein purification.

Protein purification of PTPs for enzymatic assays was conducted at 4 °C. Frozen cell pellets were lysed by sonication in 40 mL cold lysis buffer (50 mM Tris-HCl, pH 8.0, 150 mM NaCl, 5 mM imidazole, and 1 mM PMSF) per liter cell pellet. Cell lysates were clarified by centrifugation using a Beckman JA-18 rotor for 15 min at 5308 x g. The supernatant was incubated with HisPur Ni-NTA resin (Thermo Scientific) for 2 h and then packed onto a column and washed with 50 resin volume of buffer A (50 mM Tris-HCl, pH 8.0, 500 mM NaCl, 5 mM imidazole). The His-tagged proteins were eluted with Buffer B (50 mM Tris-HCl, pH=8.0, 500 mM NaCl, 300 mM imidazole). Pooled His-protein-containing fractions were concentrated, loaded onto a HiLoad 26/600 Superdex 75 column (GE Healthcare Biosciences), and eluted with storage buffer (50 mM Tris-HCl, pH 8.0, 150 mM NaCl, 1 mM DTT, 10% glycerol). Proteins used for inhibition assays were purified using Ni-NTA resin (Qiagen) followed by size exclusion column chromatography (ÄKTA pure, Cytiva), and the purity was determined to be >95% by SDS-PAGE and Coomassie staining. The protein was aliquoted and stored at −80 °C.

**High-Throughput Screening of SHP1 Covalent Inhibitors:** The cysteine-focused electrophilic fragment library was assembled through purchasing from Enamine and composed of 4,611 compounds. The HTS was performed using an adapted protocol as previously reported (Qu et al., 2024). The DMSO stocks of compounds were transferred into NUNC 384-well plates (ThermoFisher Cat. 242765) using the Echo 550 Liquid Handler. PTPs (SHP1, SHP2, PTP1B, or PTPN22) were pre-diluted in DMG buffer (50 mM 3,3-dimethylglutaric acid, pH 7.0, 1 mM EDTA, 18 mM NaCl, 0.01% Triton X-100) to a final concentration of 400 nM and dispensed into corresponding wells by MultiDrop Dispenser to incubate with compounds of 500 µM concentration. Compounds and proteins were incubated for 30 minutes at room temperature, followed by the addition of 40 µL of pNPP pre-diluted in DMG buffer (final concentration 40 mM). The enzymatic reaction was allowed to proceed for 30 seconds (PTP1B), 2 minutes (SHP1 and SHP2), or 10 minutes (PTPN22) before quenching with 40 µL of 5-N NaOH. OD_405_ was determined by a SpectraMax Microplate Reader, and background subtraction and percent inhibition were calculated using GraphPad 9.6.2.

**Phosphatase Activity Assay:** To determine the inhibition kinetics of acquired hits, repurchased compounds from Enamine were serially diluted in a 96-well plate followed by the addition of pNPP at *K*_m_ concentrations of corresponding proteins. The reaction was initiated by the addition of 20 nM proteins, and the reaction progression was monitored at OD_405_ using a CLARIOstar Plus Microplate Spectrophotometer (BMG Labtech) for 60 minutes. After background subtraction, the obtained progress curves were fitted to the equation A = v_0_(1-e^-kt^)/k, where A is the absorbance at 405 nm, v_0_ is the initial rate, k is the k_obs_ of the corresponding reaction. The acquired k_obs_ was then plotted against inhibitor concentrations, which were fitted to k_obs_ = k_inact_[I]/(K_I_+[I]), where k_inact_ is the maximum rate of inactivation, [I] is the inhibitor concentration, and K_I_ is the reversible binding affinity of the compound to the protein. Data were fitted using Prism GraphPad 10.1.1.

To further confirm the time-dependent inhibition and determine the IC_50_ of acquired hits, repurchased compounds from Enamine were serially diluted in a 96-well plate, followed by the addition of proteins at 200 nM. After 10-minute and 60-minute preincubation, 2 µL of the mixture was transferred into 100 µM 6,8-Difluoro-4-Methylumbelliferyl Phosphate (DiFMUP, Invitrogen, cat# D6567) at a final volume of 200 µL. The reaction was allowed to proceed for 5 minutes and quenched by the addition of 40 µl of a 160 µM solution of bpV(Phen) (Sigma-Aldrich, cat# SML0889). The fluorescence signal was measured using a CLARIOstar Plus Microplate Spectrophotometer (BMG Labtech) using excitation and emission wavelengths of 340 nm and 450 nm, respectively. Data were fitted using Prism GraphPad 10.1.1.

**Intrinsic Reactivity and Stability Characterization:** To determine compound half-life in GSH, 10 µM compound was mixed with 1 mM GSH in DMG buffer, and the compound consumption was monitored using single ion monitoring (SIMS) on an Agilent Technologies 6470 series, triple quadrupole LC-MS for 48 hours. The area under the curve (AUC) of each measurement was normalized to 10 µM compounds to calculate the percent remaining. Data were fitted using Prism GraphPad 10.1.1.

**Mass Spectrometry Analysis:** For intact protein analysis, to unfold protein and quench protein-ligand reactivity, 10 µL of treated or untreated 10 µM SHP1 was diluted in 40 µL of 80% (v/v) formic acid initially, followed by 150 µL of LC-MS buffer (50/50 water/acetonitrile with 0.1% formic acid). 20 - 25 pmol per sample was analyzed by LC/MS (Agilent 1260 Infinity II with a ZORBAX Rapid Resolution High Definition 300Å Stable Bond C3, 2.1 x 100 mm, 1.8 µm column) attached to an Agilent 6129 quadrupole mass spectrometer in positive ion mode. The column was held at 45 °C. Mobile solution A was 0.1% formic acid in water, and mobile phase B was 0.1% formic acid in acetonitrile. The gradient used was held at 10% B for 5 min, increased linearly to 80% B for 15 min, and then held at 80% B for 5 min. The mass data was collected at a range of 600 - 1500 m/z. Raw data was processed using MestReNova. For all samples, the deconvoluted mass was focused on charged state 49, m/z range of 745 – 760 Da, and the deconvoluted mass range from 36500 – 37125 Da. An abundance threshold of 15% ± 5% and tolerance of 25 ± 5 ppm was set for deconvolution. For each deconvolution, the highest deconvoluted mass intensity was utilized as the mass pivot point to identify reacted SHP2 adducts. To calculate the percent SHP1 reacted, the relative abundance intensity of reacted SHP1 was divided by the total relative abundance intensity of unreacted and reacted SHP1.

For chemical proteomics studies, Cells were placed in 1 mL urea lysis buffer (100 mM Tris-HCl, pH 8, 8 M urea) and sonicated on ice for three 1-min rounds at 15% amplitude. Lysates were then centrifuged at 12,000 g, 4 ^o^C for 15 min. Total protein concentrations were determined using the bicinchoninic acid (BCA) protein assay kit (Thermo Scientific Pierce, Rockford, IL). 100 μg of proteins was reduced with 10 mM DTT for 45 min at 37 °C. Proteins were then biotinylated with 30 mM maleimide-biotin (Mal-Biotin) (Sigma-Aldrich). After a 30-min rotation at room temperature, the unreacted reagent was quenched with 10 mM DTT for an additional 30 min. Proteins were precipitated by 10% trichloroacetic acid (TCA) and centrifuged at 20,000 g for 15 min at 4 °C. The pellet was washed with ice-cold 5% TCA and then washed with 95% ethanol for two times. Proteins were resuspended in 1.5 M urea solution. Trypsin was added at 1:50 (enzyme: protein) and digested overnight at 37 °C. Peptides were then desalted and vacuum centrifuged to dryness.

Mal-biotin labeled peptides were diluted in 1 mL of PBS with 50 μL of High-Capacity NeutrAvidin slurry (Thermo Scientific). After incubation at 4^o^C for 2h, the sample was washed with 1 mL of PBS twice, 50 mM ammonium bicarbonate with 20% methanol once, and eluted with 50% ACN with 0.4% trifluoroacetic acid (TFA). Peptides were then vacuum centrifuged to dryness. The peptide samples were analyzed by using an Evosep One LC coupled to a timsTOF HT mass spectrometer (Bruker). The standard 40 SPD (sample per day) method was utilized in the Evosep One LC, in which a 15 cm × 75 μm reverse-phase column packed with 1.5 μm C18 beads (PepSep) was used. The analytical column was connected with a fused silica emitter (10 μm ID; Bruker Daltonics) inside a nanoelectrospray ion source (Captive Spray source; Bruker). The mobile phases comprised 0.1% FA as buffer A and 0.1% FA in 99.9% acetonitrile (ACN) as buffer B. Details of the liquid phase gradient for the Evosep One LC system were proprietary.

The tipstaff HT instrument was operated in a data-dependent acquisition (DDA)-PASEF scan mode consisting on 10 MS/MS PASEF scans per topN acquisition cycle, with an accumulation time of 100 ms and a ramp of 100 ms. MS and MS/MS spectra were acquired in an m/z range from 300 to 1200 and in an ion mobility range (1/K0) from 0.60 to 1.60 V s/cm^2^, selecting precursor ions for the MS/MS PASEF scans from a previous TIMS-MS scan. The collision energy was programmed as a function of ion mobility, following a straight line from 20 eV for 1/K_0_ of 0.6 to 59 eV for 1/K_0_ of 1.6.

The acquired raw mass spectra were processed with MSfragger software version 20.0 against the human protein database from UniProt (https://openprot.org/). The parameters were set up as follows. Enzyme: trypsin (full) with maximum missed cleavages was 2; variable modifications: oxidation (Met), N-acetylation, Mal-biotin labeled cysteine (451.1889 Da); precursor mass tolerance was set to 20 ppm; fragment mass tolerance: 0.05 Da; false discovery rate (FDR) was defined as 1%. Modified peptide identifications with a search score greater than 40, a delta score greater than 6, and a localization probability >0.8 were allowed.

**Immunoblotting:** Cultured cells were lysed with ice-cold lysis buffer (50 mM Tris, pH 8.0, 150 mM NaCl, 10% Glycerol, 1% Triton-X-100) supplied with phosphatase inhibitor (Bimake) and protease inhibitor mixture (Roche Applied Science). Equal amounts of protein were resolved by SDS-PAGE, transferred to a nitrocellulose membrane, and subjected to immunoblotting. Antibodies used in this study include anti-following proteins: pLck pY394 (Bio-Techne #MAB7500, 1:3000), Lck (Santa Cruz #sc-433, 1:3000), pERK1/2 pT202/pY204 (Cell Signaling Technology #4370), ERK1/2 (Cell Signaling Technology Cat# 4696), p-p65 pS536 (Cell Signaling Technology #3033, 1:3000), p65 (Cell Signaling Technology #8242, 1:3000), p-PLCγ 1 pY783(Cell Signaling Technology #2821s, 1:1000), PLCγ 1 (Cell Signaling Technology #5690p, 1:1000), GAPDH (Cell Signaling Technology #97166, 1:5000), Anti-rabbit IgG, HRP-linked (Cell Signaling Technology #7074, 1:3000) and Anti-mouse IgG, HRP-linked (Cell Signaling Technology#7076, 1:3000).

**Cell Permeability:** The cell permeability of tested compounds was determined with Corning® BioCoat® Pre-coated PAMPA Plate System as per the manufacturer’s guidance. Specifically, the Compound solutions were prepared by diluting 10 mM DMSO stock solutions in PBS (in most cases, we used a final concentration of 200 µM). The compound solutions were added to the wells (300 µL/well) of the receiver plate, and PBS was added to the wells (200 µL/well) of the pre-coated filter plate. The filter plate was then coupled with the receiver plate, and the plate assembly was incubated at room temperature without agitation for five hours. At the end of the incubation, the plates were separated, and 150 µL solution from each well of both the filter plate and the receiver plate was sampled. The final concentrations of compounds in both donor wells and acceptor wells were analyzed by LC-MS analysis. Permeability of the compounds was calculated using the following formula:

Permeability (cm/s): Pe = {-ln[1-CA(t)/Ceq]}/[A*(1/VD+1/VA)*t] , whereas:

A = filter area (0.3 cm2), VD = donor well volume (0.3 mL), VA = acceptor well volume (0.2 mL), t = incubation time (seconds), CA(t) = compound concentration in acceptor well at time t,

CD(t) = compound concentration in donor well at time t, and Ceq = [CD(t)*VD+CA(t)*VA]/(VD+VA)

**Cytotoxicity:** HEK293, MC38, EO771, RAW264.7 cells in DMEM media [supplemented with 10% (v/v) fetal bovine serum and penicillin (100 units/ml)/streptomycin (100 μg/ml)] and Jurkat T cells in RPMI 1640 media [supplemented with 10% (v/v) fetal bovine serum and penicillin (50 units/ml)/streptomycin (50 μg/ml)] were dispensed in clear flat-bottom 96-well plates at the density of 5,000 cells per well and culture for 16 hours under 37 °C with 5% CO_2_. M029 of different concentrations or DMSO was added to each well, followed by additional incubation for 24 hours. All wells were then treated with 5 µL of CCK8 reagent (Abcam, #ab228554) followed by 4 hours of incubation. OD_460_ of the reaction mixture was detected using a Spectra MAX340 microplate spectrophotometer (Molecular Devices) and was used to determine the percent growth inhibition of M029 on each cell line. Data were fitted using Prism GraphPad 10.1.1.

**Animal Studies**

All the in vivo studies were performed under an animal protocol (1511001324) approved by the Institutional Animal Care & Use Committee of Purdue University, in accordance with the recommendations in the Guide for the Care and Use of Laboratory Animals of the National Institutes of Health. For PK study, C57BL6 female mice (25-30g body weight) were injected intraperitoneally, orally, or intravenously with 25 mg/kg M029 dissolved in 0.4 ml saline. Blood samples were collected through the tail vein at the indicated time points after injection. Isoflurane was used as an anesthetic. All blood samples were centrifuged at 1,500 g for 5 minutes, and plasma was separated and stored at –80°C until analysis by a validated method based on reversed-phase liquid chromatography coupled to mass-spectrometric detection (LC/MS) using a previously published procedure.^169^ For the MC38 syngeneic tumor study, 12-week-old C57BL6 female mice were injected subcutaneously with 106 MC38 cells in the right and left flanks for tumor growth. When tumors reached an average volume of 100 mm^3^ mice were tumor size-matched and randomly assigned to different experimental groups for experiments. Four mice with eight tumors were used in each treatment group. Mice were injected orally with saline or 25 or 100 mg/kg M029 daily. Tumor sizes and animal weights were measured twice per week. Tumor volume (mm^3^) = (length X width^2^)/2. At the end of the experiments, mice were euthanized, and tumors were collected for analysis. Among eight tumors collected from each group, four homogenous samples and six sections were prepared for Western blots and histology analysis.

**Flow cytometry**

For immune cell profiling in the tumor microenvironment, tumor samples were first reduced to single-cell suspensions by compressing the tumors through a 70 µm filter (Falcon, #352350) into cold 1× PBS supplemented with 5 mM EDTA using the plunger of a syringe (HSW, #4010-200V0). Samples were strained through a second filter and then re-suspended in cold blocking buffer made of 5% BSA, 0.5 mM EDTA, and a 1:1000 dilution of CD16/32. Samples were then incubated with following fluorophore-conjugated antibodies. CD4-488 (Biolegend #100423), CD8a-PE (Biolegend #100708), CD45-PeCy7 (Biolegend #103114), CD25-BV605 (Biolegend #102035), CD44-647 (Biolegend #103039), CD19-488 (Biolegend #115524), NK1.1-647 (Biolegend #108719), CD3-PeCy5 (Biolegend #100273), CD8-BV711 (Biolegend #100747), CD4-PeCy5 (Biolegend #100409), CD69-FITC (Biolegend #104505), FoxP3-488 (Biolegend #126405), Perforin-APC (Biolegend #154303), CD62L-BV605 (Biolegend #104437), TIM3-PerCP/Cy5.5 (Biolegend #134011), PD1-647 (Biolegend #109118), CD11b-BV711 (Biolegend #101241), CD206-FITC (Biolegend #141704), F4/80-647 (Biolegend #123122), CD86-PE (Biolegend #159203), CD11c-BV421 (Biolegend #117329), Ly6G-488 (Biolegend #127625), CD11b-APC (Biolegend #101211), Ly6C-PE (Biolegend #128007), F4/80-BV711 (Biolegend #123147), IL2-BV421 (Biolegend #503825), IFNγ-BV605 (Biolegend #505839), TNFα-APC (Biolegend #506307). The following antibodies were used for depletion studies: anti-CD8α (BioXCell #BE0061) and anti-NK1.1 (BioXCell #BE0036). Samples were incubated with antibodies at a 1:600 dilution in blocking buffer on ice for two hours. After staining, samples were washed and re-suspended in 5% BSA, 0.5 mM EDTA solution, then added to a 5 mL polystyrene tube for analysis (Falcon, #352235). Sample data was acquired using a BD LSRFortessa Cell Analyzer, and results were analyzed using FlowJo.

**Quantification and statistical analysis**

Statistical analyses were performed with GraphPad Prism software 10.4.2 through the student t test or the one-way ANOVA test using Turkey post-hoc comparison according to the experiment. Error bars in figures indicate average ± standard error of the mean (SEM). *p<0.05, **p<0.01, ***p<0.001, and ****p<0.0001 were considered statistically significant.

**Compound synthesis**

As depicted in Scheme S1, Z57117023 was synthesized via the sulfonylation of 2-aminothiazole by the commercially available *tert*-butyl (4-(chlorosulfonyl)phenyl)carbamate, followed by the removal of the Boc protecting group and the subsequent amide formation with chloroacetyl chloride.

Scheme S1. Synthesis of Z57117023

Reagents and conditions: (a) pyridine, toluene, 65 °C, overnight. (b) (i) TFA, DCM, rt, overnight. (ii) chloroacetyl chloride, DCM, 0 °C then rt, 6 h.

Compounds M029 and M054 were synthesized using the same methods as the hit Z57117023 with the corresponding amine substrates and chloroacetyl chloride or acetyl chloride. The compound M037 was synthesized via the sulfonylation of 2-aminothiophene-3-carbonitrile by 3-chloro-4-nitrobenzenesulfonyl chloride, followed by the reduction of the nitro group and the subsequent formation of the chloroacetamide with chloroacetyl chloride (Scheme S2).

Scheme S2. Synthesis of M037^a^

*^a^*Reagents and conditions: (a) pyridine, toluene, 65 °C, overnight. (b) SnCl_2_, 50 °C, overnight. (c) chloroacetyl chloride, DCM, 0 °C then rt, overnight.

*Synthesis of intermediate* ***1****.*

*tert*-Butyl (4-(chlorosulfonyl)phenyl)carbamate (1.0 eq.) and the amine of interest (1.5 eq.) were suspended in toluene, and then pyridine (3.0 eq.) was added. The reaction mixture was stirred at 65 °C overnight, and the reaction completion was confirmed by LC-MS. The solvent was evacuated in vacuo, and the residue was purified by flash chromatography (Hexane/Ethyl acetate, 0-60%) to deliver corresponding carbamates. Yields range between 30% and 65%.

*tert*-butyl (4-(*N*-(thiazol-2-yl)sulfamoyl)phenyl)carbamate (**1a**).

^1^H NMR (500 MHz, DMSO) δ 9.72 (s, 1H), 7.66 (m, 2H), 7.55 (m, 2H), 7.21 (d, *J* = 5.0 Hz. 1H), 6.78 (d, *J* = 4.5 Hz, 1H), 1.46 (s, 9H). LC/MS m/z calculated [M+H]^+^ 356.07, found 356.10.

*tert*-butyl (4-(*N*-(3-cyanothiophen-2-yl)sulfamoyl)phenyl)carbamate (**1b**).

^1^H NMR (500 MHz, DMSO) δ 9.88 (s, 1H), 7.62 (s, 3H), 7.35 (d, *J* = 5.9 Hz, 2H), 7.18 (d, *J* = 5.8 Hz, 2H), 1.47 (s, 9H). LC/MS m/z calculated [M-H]^+^ 378.07, found 378.15.

*tert*-butyl (2-chloro-4-(*N*-(3-cyanothiophen-2-yl)sulfamoyl)phenyl)carbamate (**1c**).

^1^H NMR (500 MHz, DMSO) δ 8.22 (d, *J* = 8.4 Hz, 1H), 7.99 (d, *J* = 1.8 Hz, 1H), 7.87 (dd, *J* = 8.5, 1.9 Hz, 1H), 7.09 – 7.01 (m, 2H). LC/MS m/z calculated [M-H]^+^ 341.95, found 341.83.

*Synthesis of intermediate* ***2****.*

The nitrobenzene generated from step (a) (1.0 eq.) was dissolved in MeOH, followed by the addition of SnCl_2_ (3.0 eq.), and the reaction mixture was stirred for 16 hours at 50 °C. The reaction completion was confirmed using LC-MS, and residual SnCl_2_ was precipitated by adding saturated NaHCO_3_ solution. The mixture was filtered, and the filtrate was collected. The solvent of the filtrate was evaporated in vacuo, followed by purification using a reverse-phase flash column (MeOH/H_2_O, 0-70%) to deliver corresponding anilines. Yield 60%.

4-amino-3-chloro-*N*-(3-cyanothiophen-2-yl)benzenesulfonamide (**2**).

^1^H NMR (500 MHz, DMSO) δ 7.86 (d, *J* = 2.1 Hz, 1H), 7.68 (dd, *J* = 9.1, 2.1 Hz, 1H), 7.32 (d, *J* = 5.5 Hz, 1H), 7.12 (d, *J* = 5.7 Hz, 1H), 6.94 (d, *J* = 9.1 Hz, 1H), 5.22 (s, 2H). LC/MS m/z calculated [M-H]^+^ 311.97, found 311.95.

*Synthesis of Z57117023***,** *M029, M037, and M054.*

The aniline **1** or **2** (1.0 eq.) was dissolved in anhydrous DCM, followed by dropwise addition of chloroacetyl chloride (3.0 eq.) or acetyl chloride at 0 °C. The reaction mixture was stirred for 6 hours at room temperature. Completion of the reaction was confirmed by LC-MS. The reaction was quenched by DI water at 0 °C. The residual DCM was evaporated in vacuo, and the mixture was loaded to and purified by reverse-phase flash column (MeOH/H_2_O, 0-70%). Yield ranging between 35% and 70%.

2-chloro-*N*-(4-(N-(thiazol-2-yl)sulfamoyl)phenyl)acetamide (**Z57117023**).

^1^H NMR (500 MHz, DMSO) δ 10.59 (s, 1H), 7.75 (m, 2H), 7.70 (m, 2H), 7.22 (d, *J* = 5.0 Hz, 1H), 6.79 (d, *J* = 5.0 Hz, 1H), 4.26 (s, 2H). LC/MS m/z calculated [M-H]^+^ 329.99, found 329.82.

2-Chloro-*N*-(4-(*N*-(3-cyanothiophen-2-yl)sulfamoyl)phenyl)acetamide (**M029**).

^1^H NMR (500 MHz, DMSO) δ 10.72 (s, 1H), 7.81 – 7.75 (m, 3H), 7.74 – 7.68 (m, 2H), 7.37 (d, *J* = 5.8 Hz, 1H), 7.19 (d, *J* = 5.8 Hz, 1H), 4.29 (s, 2H). ^13^C NMR (125 MHz, DMSO) δ 165.89, 148.69, 143.44, 133.07, 128.91, 127.29, 124.69, 119.65, 114.25, 104.67, 44.04, 31.16. LC/MS m/z calculated [M-H]^+^ 353.98, found 353.93.

2-Chloro-*N*-(2-chloro-4-(*N*-(3-cyanothiophen-2-yl)sulfamoyl)phenyl)acetamide (**M037**).

^1^H NMR (500 MHz, DMSO) δ 9.55 (s, 1H), 9.44 (s, 1H), 8.18 (d, *J* = 9.2 Hz, 1H), 7.96 (d, *J* = 2.1 Hz, 1H), 7.76 (dd, *J* = 9.2, 2.1 Hz, 1H), 7.26 (s, 1H), 7.11 (d, *J* = 5.7 Hz, 1H), 4.22 (s, 2H). LC/MS m/z calculated [M-H]^+^ 388.95, found 388.86.

*N*-(4-(*N*-(3-cyanothiophen-2-yl)sulfamoyl)phenyl)acetamide (**M054**).

The compound was prepared following general methods using the same starting materials as to prepare **M029**. The aniline acquired reacted with acetyl chloride instead of chloroacetyl chloride, following the same stoichiometry of the general method to deliver compound **M054**. ^1^H NMR (500 MHz, DMSO) δ 10.35 (s, 1H), 7.77 – 7.70 (m, 2H), 7.69 – 7.61 (m, 2H), 7.38 – 7.29 (m, 1H), 7.16 (d, *J* = 5.7 Hz, 1H), 6.60 – 6.54 (m, 1H), 2.07 (s, 3H). LC/MS m/z calculated [M-H]^+^ 320.02, found 320.13.

**References**

Qu, Z., Krabill, A. D. and Zhang, Z.-Y. (2024). High throughput discovery and characterization
 of covalent inhibitors for protein tyrosine phosphatases. Methods Mol. Biol*.* *2743*, 301-
 316.

**NMR Spectra**

^1^H NMR of **M029**

^13^C NMR of **M029**

^1^H NMR of **M054**
